# 1,4-Diazatriphenylene and Its Hetero-Fused Analogs: Synthesis and Applications

**DOI:** 10.3390/molecules31122197

**Published:** 2026-06-22

**Authors:** Egor V. Verbitskiy, Elizaveta M. Krynina, Yuriy A. Kvashnin, Valery N. Charushin

**Affiliations:** 1 I. Ya. Postovsky Institute of Organic Synthesis, Ural Branch of the Russian Academy of Sciences, S. Kovalevskaya Str., 22, 620137 Ekaterinburg, Russia; 2 Department of Organic and Biomolecular Chemistry, Ural Federal University, Mira St. 19, 620002 Ekaterinburg, Russia

**Keywords:** 1,4-diazatriphenylenes, polycyclic systems, organic semiconductors, biological activity

## Abstract

This review highlights the recent advances in the synthesis of 1,4-diazatriphenylenes and their various structural analogs. It focuses on several methodologies, including condensation reactions and intramolecular cyclizations of 2,3-di(het)aryl-substituted pyrazine derivatives. These methods exploit either oxidative photocyclization (the Mallory reaction), intramolecular cyclodehydrogenation (the Scholl reaction), or intramolecular S_N_^H^ reactions (nucleophilic aromatic substitution of hydrogen) involving 2-*bis*(het)aryl-substituted 1,4-diazine derivatives. Additionally, the review explores the potential applications of these compounds as fluorescent and/or semiconducting materials in organic electronics, as well as their role in coordination chemistry and biological issues. It summarizes the literature from 2018 to March 2026, complementing the data discussed in our previous review.

## 1. Introduction

Over the past three decades, polycyclic aromatic hydrocarbons have gained widespread attention due to their use in materials chemistry, organic electronics, and the design of chemosensors and molecular probes for medical applications [1,2]. (Aza)triphenylenes are tetracyclic structures representing the smallest examples of benzenoid polycyclic aromatic hydrocarbons. (Aza)triphenylene derivatives have gained significant attention in supramolecular and heterocyclic chemistry due to their rigid, planar, and conjugated structures, as well as their self-assembling properties [3,4,5]. A remarkable compound in this area is 1,4-diazatriphenylene (also known as dibenzo[*f*,*h*]quinoxaline), along with its fused analogs. These polycyclic nitrogen-containing heteroaromatic compounds are characterized by fully conjugated frameworks that are enforced to be planar and exhibit threefold rotational symmetry. They have become the key building blocks in advanced materials chemistry. These structurally rigid systems demonstrate exceptional electron delocalization and supramolecular ordering, making them highly suitable for applications in optoelectronic devices, functional coordination polymers, and energy storage systems [3,4,5,6].

**Figure 1:**
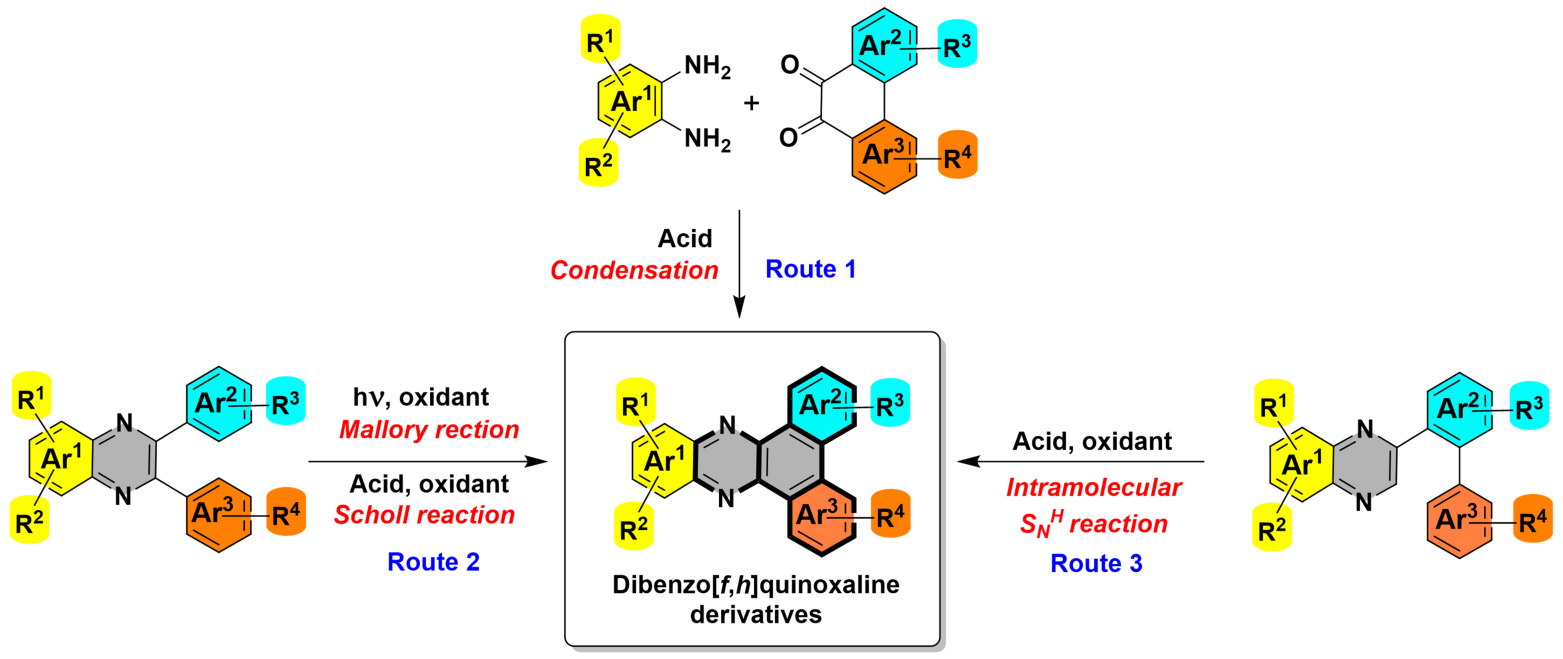
Synthetic approaches to 1,4-diazatriphenylene derivatives and their heteroanalogs.

There are several key routes to construct the 1,4-diazatriphenylene scaffold, based on condensation reactions (*Route 1*) [4,5], intramolecular cyclizations of 2,3-di(het)aryl-substituted pyrazines using oxidative photocyclization (the Mallory reaction) [6,7] or intramolecular cyclodehydrogenation catalyzed by chemical oxidants under acidic conditions via the Scholl reaction (*Route 2*) [8,9], or intramolecular S_N_^H^ reactions (nucleophilic aromatic substitution of hydrogen) of 2-*bis*(het)aryl-substituted 1,4-diazine derivatives (*Route 3*) [10,11] (Figure 1).

The most common method to obtain 1,4-diazatriphenylene derivatives is based on the condensation of 1,2-diamines with substituted phenanthrene-9,10-diones and their heteroanalogs (see Figure 1, *Route 1*). However, a significant limitation of this method is due to difficulties in obtaining and structural identification of asymmetric 1,4-diazatriphenylene derivatives when R^1^ ≠ R^2^ and R^3^ ≠ R^4^.

**Figure 2:**
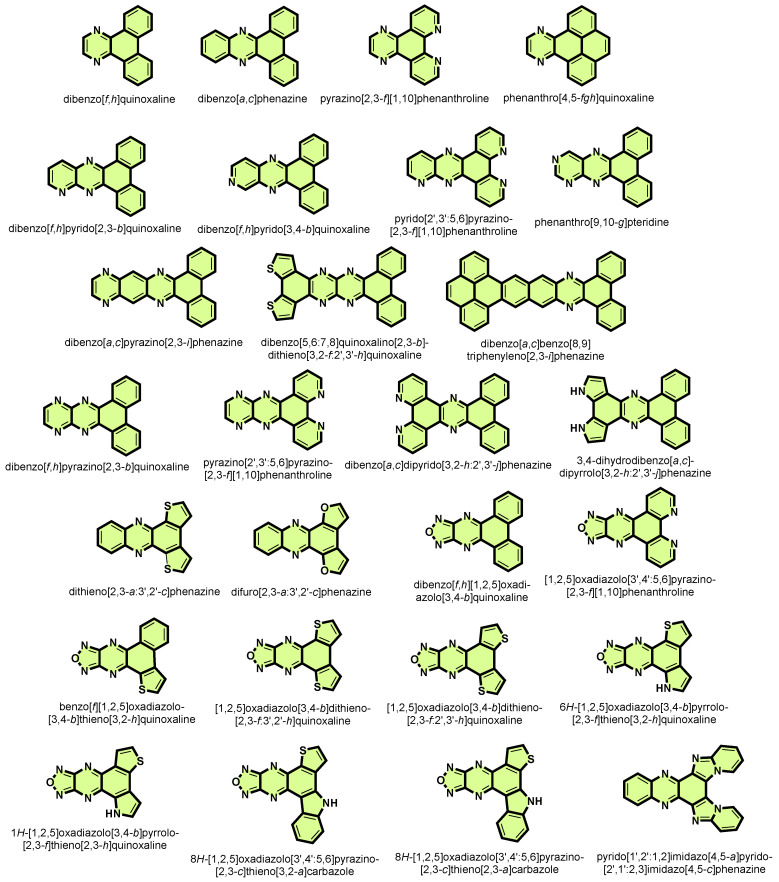
Structures of various 1,4-diazatriphenylenes and their hetero-fused analogs, which are discussed in this review.

Due to their broad applications in various fields, continuous efforts are being made to develop new, convenient, and efficient synthetic approaches to a variety of 1,4-diazatriphenylene derivatives. Based on existing practices and relevant research areas, two comprehensive reviews, focusing on modern methods for synthesizing diazatriphenylenes, were published in 2017 and 2018 [4,5]. In this review article, we wish to discuss the topic by primarily examining the literature data published from 2018 to the present. Figure 2 illustrates the structures of various 1,4-diazatriphenylenes and their condensed analogs, which are covered in this review.

## 2. Synthesis Using Cyclocondensation Reactions

**Scheme 1:**
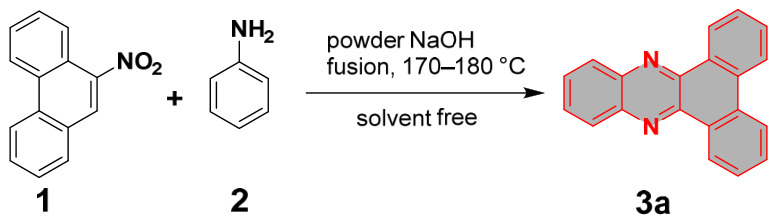
Synthesis of dibenzo[*a*,*c*]phenazine (**3a**).

The first derivative of 1,4-diazatriphenylene, dibenzo[*a*,*c*]phenazine (**3a**), was synthesized in 1951 through the condensation of 9-nitrophenanthrene (**1**) with aniline (**2**) on heating without solvent at 170–180 °C in the presence of powdered NaOH as a catalyst (Scheme 1) [4].

**Scheme 2:**
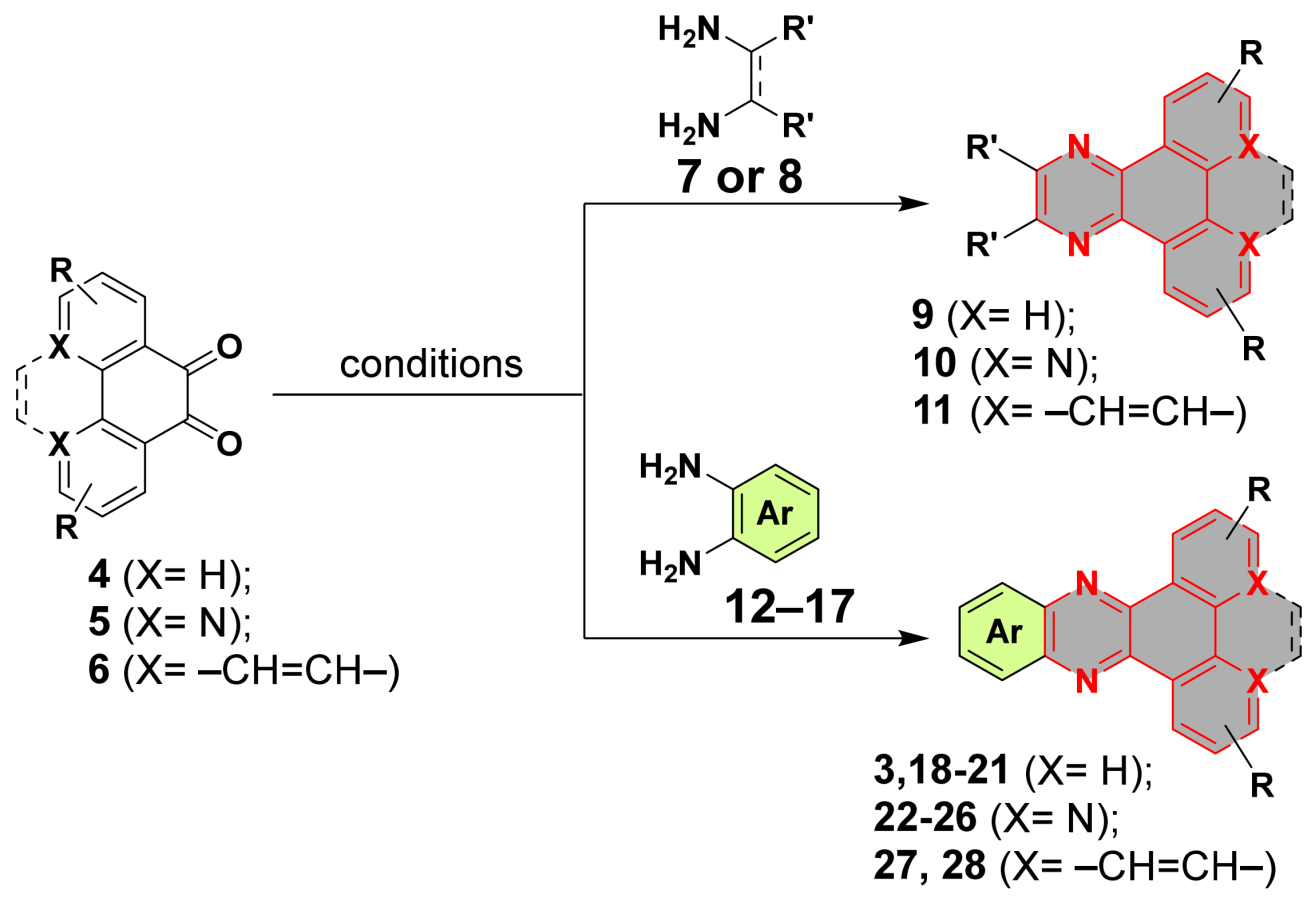
Synthesis of dibenzo[*a*,*c*]phenazine (**9**), pyrazino[2,3-*f*][1,10]phenatroline (**10**), phenanthro[4,5-*fgh*]quinoxaline (**11**), dibenzo[*f*,*h*]quinoxaline (**3**, **18**–**21**), pyrazino[2,3-*f*][1,10]phenatrolines (**22**–**26**), and phenanthro[4,5-*fgh*]quinoxalines (**27**, **28**).

As noted above, currently the most widespread method used for the synthesis of 1,4-diazatriphenylene derivatives and their fused analogs is the condensation of 1,2-dicarbonyl compounds, for example, derivatives of phenanthrene-9,10-dione (**4**), 1,10-phenanthroline-9,10-dione (**5**), or pyrene-4,5-dione (**6**) with the corresponding 1,2-diamines, such as derivatives of ethylenediamine (**7**, **8**) and *o*-phenylenediamine (**12**–**17**) (Scheme 2). Reaction conditions for condensations leading to derivatives of dibenzo[*a*,*c*]phenazine (**9**), pyrazino[2,3-*f*][1,10]phenatroline (10), phenanthro[4,5-*fgh*]quinoxaline (**11**), dibenzo[*f*,*h*]quinoxaline (**3**, **18**–**21**), pyrazino[2,3-*f*][1,10]phenatrolines (**22**–**26**) and phenanthro[4,5-*fgh*]quinoxalines (**27**, **28**) are presented below (Scheme 2, Table 1 and Table 2).

**Scheme 3:**
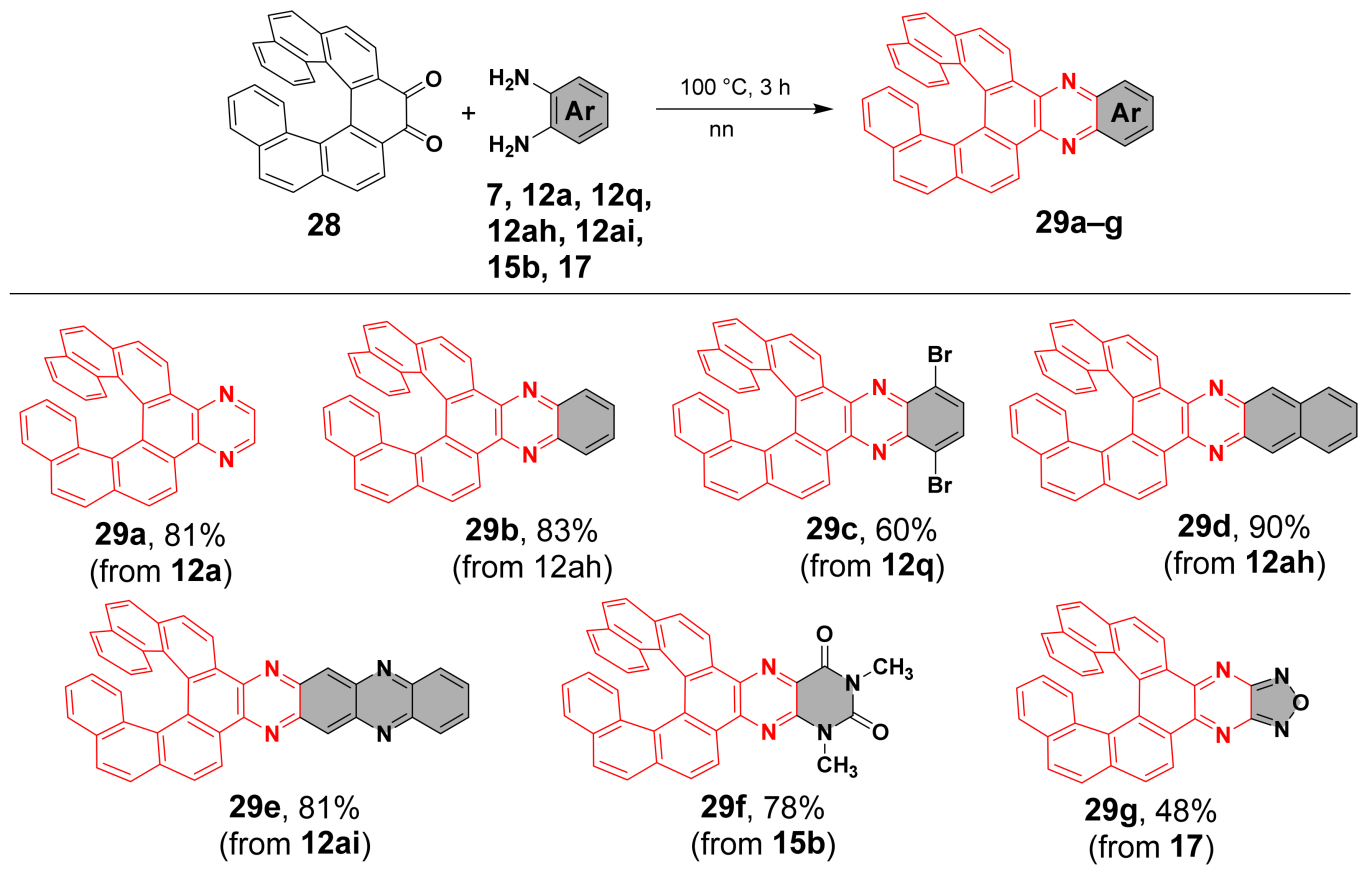
Synthesis of [7]helicenes (**29a**–**g**).

A series of various [7]helicenes containing a 1,4-diazatriphenylene moiety (171–177) were synthesized by condensation of benzo[1,2-*c*:4,3-c′]diphenanthrene-9,10-dione (**28**) with ethylenediamine (**7**) and various aromatic diamines (**12a**, **12g**, **12ah**, **12ai**, **15b**, and **17**) (Scheme 3) [182].

It is important to note that heteroanalogs of 1,4-diazatriphenylene can be obtained by condensation of 1,2-diketones with 1,2-diamines, proceeding under acidic conditions. This method has been used to obtain dithieno[3,2-*f*:2′,3′-*h*]quinoxalines and thieno[2′,3′:4,5]thieno[3,2-*f*]thieno-[2′,3′:4,5]thieno[2,3-*h*]quinoxalines [183,184,185]. Since 2018, a huge number of substituted heteroanalogs of 1,4-diazatriphenylenes (over a hundred compounds, obtained through this procedure) have been described [68,69,186,187,188,189,190,191,192,193,194,195,196,197,198,199,200,201,202,203,204,205,206,207,208,209,210,211,212,213,214,215,216,217,218,219,220,221,222,223,224,225,226,227,228,229,230,231,232,233,234,235,236,237,238,239,240,241,242,243,244,245,246,247,248,249,250,251,252,253,254,255,256,257,258,259,260,261,262,263,264,265,266,267].

## 3. Synthesis Using the Scholl Reaction

The Scholl reaction, nucleophilic aromatic substitution of hydrogen, or metal-catalyzed cross-coupling reactions are less common synthetic approaches for 1,4-diazatriphenylenes and their heteroanalogs.

**Scheme 4:**
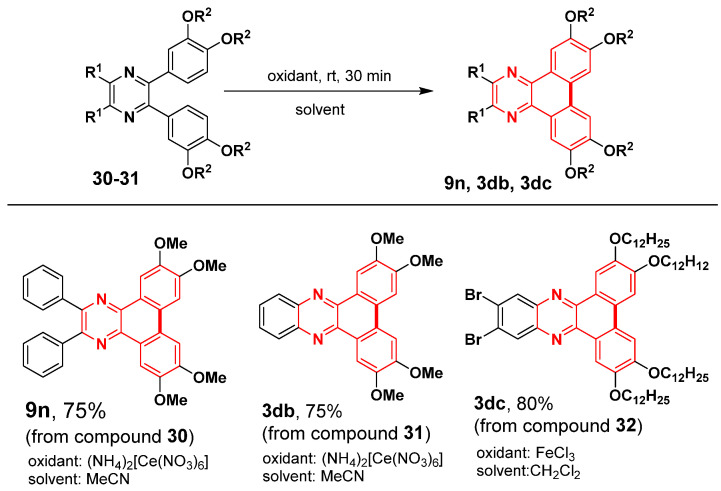
Synthesis of 1,4-diazatriphenylene derivatives **9n**, **3db**, and **3dc**.

For instance, 1,4-diazatriphenylene (**9n**) and some derivatives (**3db** and **3dc**) were synthesized via the Scholl reaction from either 2,3,5,6-tetrasubstituted pyrazine (**30**) or 2,3-disubstituted quinoxalines (**31** and **32**), as illustrated in Scheme 4 [268,269].

**Scheme 5:**
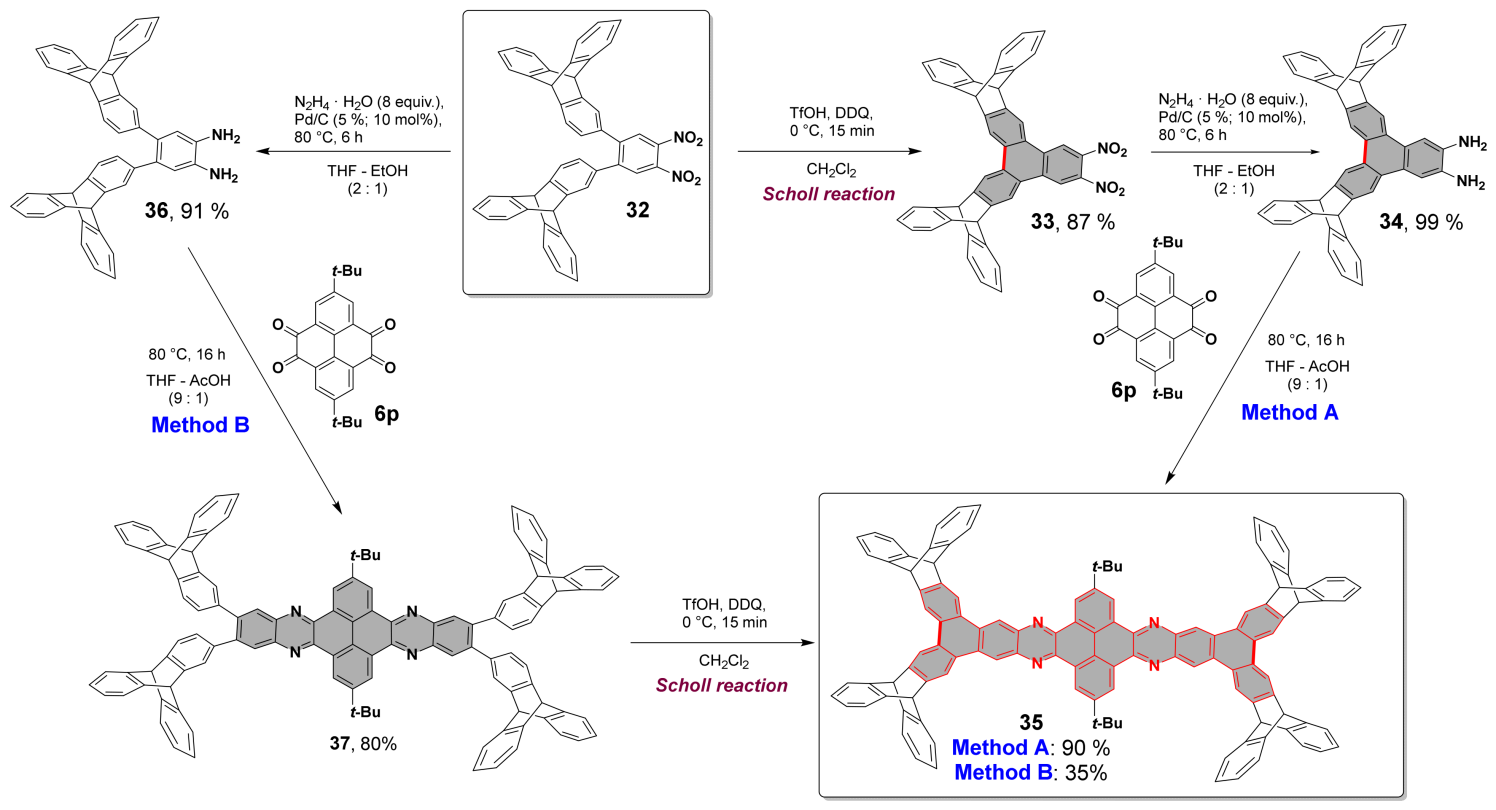
Synthesis of quinoxalino[2′,3′,9,10]phenanthro[4,5-*abc*]phenanzine (**36**).

2,2′-(4,5-Dinitro-1,2-phenylene)*bis*(9,10-dihydro-9,10-[1,2]benzoanthracene) (**32**) takes part in the Scholl reaction when treated with 2,3-dichloro-5,6-dicyano-1,4-benzoquinone (**DDQ**) and trifluoromethanesulfonic acid. The resulting polycyclic product (**33**) undergoes reduction of both nitro groups by action of hydrazine hydrate in the presence of palladium on carbon. This reduction yields the corresponding 1,2-diamino compound (**34**), which undergoes condensation with 2,7-di-*tert*-butylpyrene-4,5,9,10-tetraone (**6p**) to produce the desired 18,41-di-*tert*-butyl-5,8,13,23,28,31,36,46-octahydro-5,46:8,13:23,28:31,36-tetrakis([1,2]benzeno)heptapheno[7,8-*i*]-hepapheno[7″,8″:6′,7′]-quinoxalino[2′,3′:9,10]phenanthro[4,5-*abc*]phenazine (**35**) in 90% yield (*Method A*) (Scheme 5).

Conversely, if one starts with the dinitro derivative (**32**) and first reduces it into the corresponding 1,2-diamino compound, followed by condensation with the tetraoxo derivative (**6p**) of pyrene (**6p**) and then the Scholl reaction, the same polycyclic product (**35**) can be obtained, but in a significantly lower yield of 35% (*Method B*) (Scheme 5) [270].

A convenient synthetic approach to a variety of heteroanalogs of 1,4-diazatriphenylene is the sequential use of Scholl reactions and nucleophilic aromatic substitution of hydrogen.

**Scheme 6:**
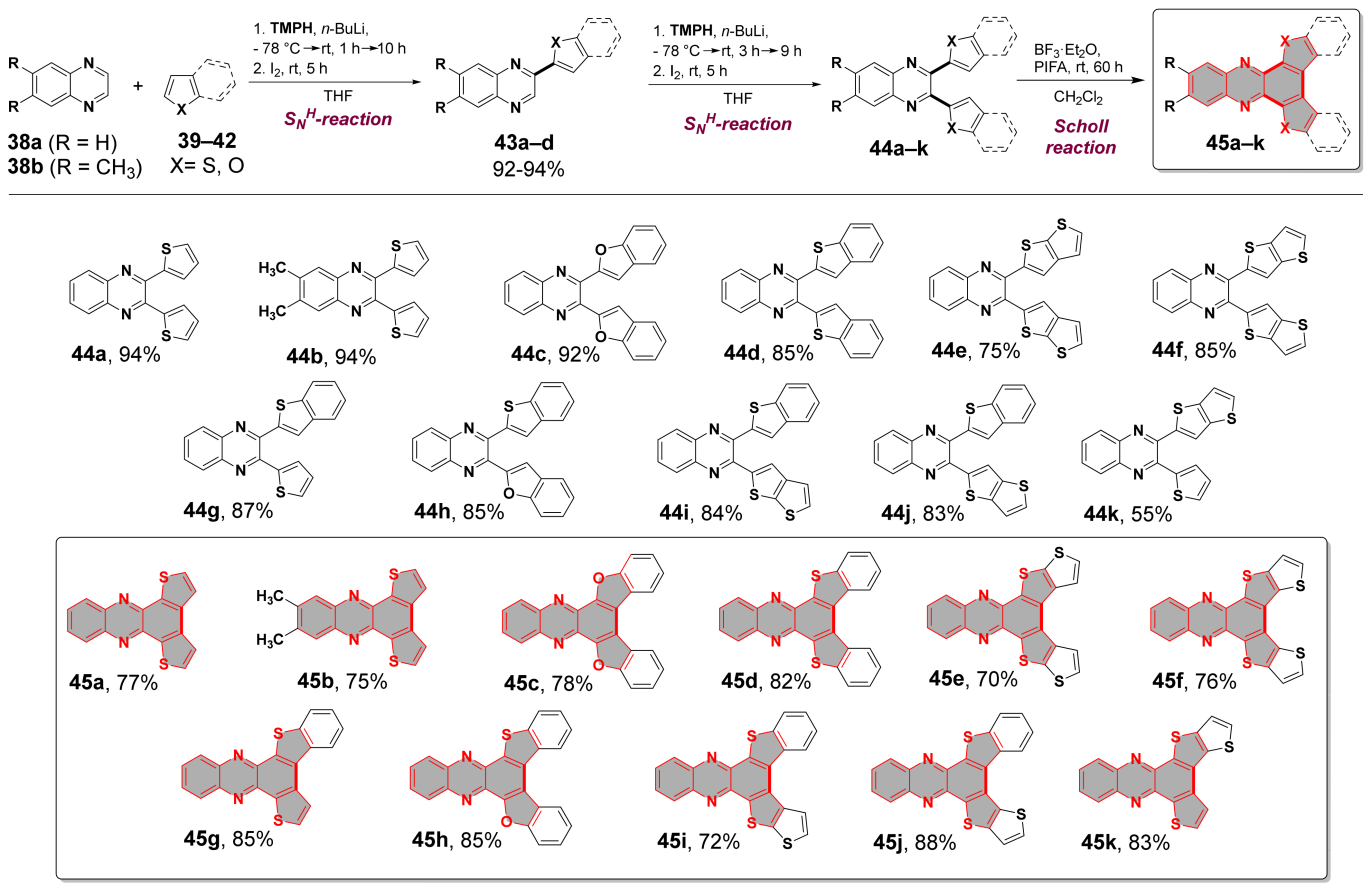
Synthesis of symmetrical (**45a**–**f**) and unsymmetrical heteroanalogs of 1,4-diazatriphenylene (**45g**–**k**).

For example, quinoxalines (**38a**,**b**) react with various thiophene derivatives and benzo[*b*]furan (**39**–**42**) in the presence of lithium 2,2,6,6,6-tetramethylpiperidine (**TMPLi**) (generated in situ during treatment of 2,2,6,6-tetramethylpiperidine (**TMPH**) with *n*-butyllithium) to afford symmetrical 2,3-di(het)aryl-substituted quinoxalines (**44a**–**k**). Derivatives **44b**–**k** were then subjected to a Scholl reaction with [*bis*(trifluoroacetoxy)iodo]benzene (**PIFA**) in the presence of boron trifluoride etherate, thus affording heteroanalogs of 1,4-diazatriphenylene (**45a**–**k**) (Scheme 6) [271].

**Scheme 7:**
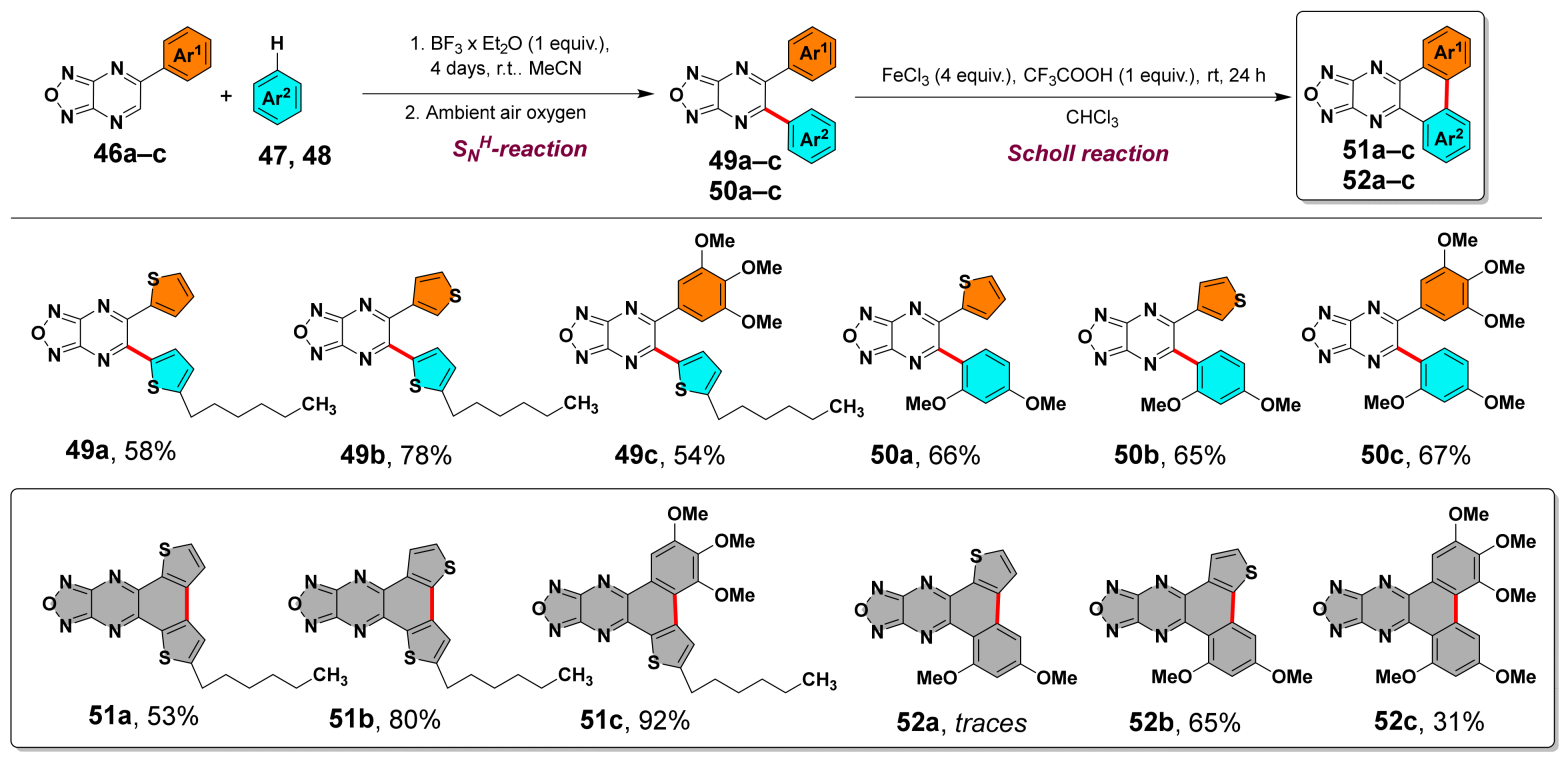
Synthesis of fused systems based on the [1,2,5]oxadiazolo[3,4-*b*]quinoxaline core (**51a**–**c** and **52a**–**c**).

[1,2,5]Oxadiazolo[3,4-*b*]pyrazine derivatives have recently gained significant attention from researchers due to their potential as high-energy compounds and their promising applications in electronics and photovoltaics [272,273,274]. It has been demonstrated that 5-(het)aryl-substituted [1,2,5]oxadiazolo[3,4-*b*]pyrazines (**46a**–**c**) can undergo a Lewis acid-catalyzed nucleophilic aromatic substitution of hydrogen (the S_N_^H^ reaction) with such *C*-nucleophiles as 2-hexyl-thiophene (**47**) and 1,3-dimethoxybenzene (**48**). This reaction leads to the formation of *ortho*-di(heteroaryl)-substituted [1,2,5]oxadiazolo[3,4-*b*]pyrazines (**51**–**52**) (Scheme 7) [275].

**Scheme 8:**
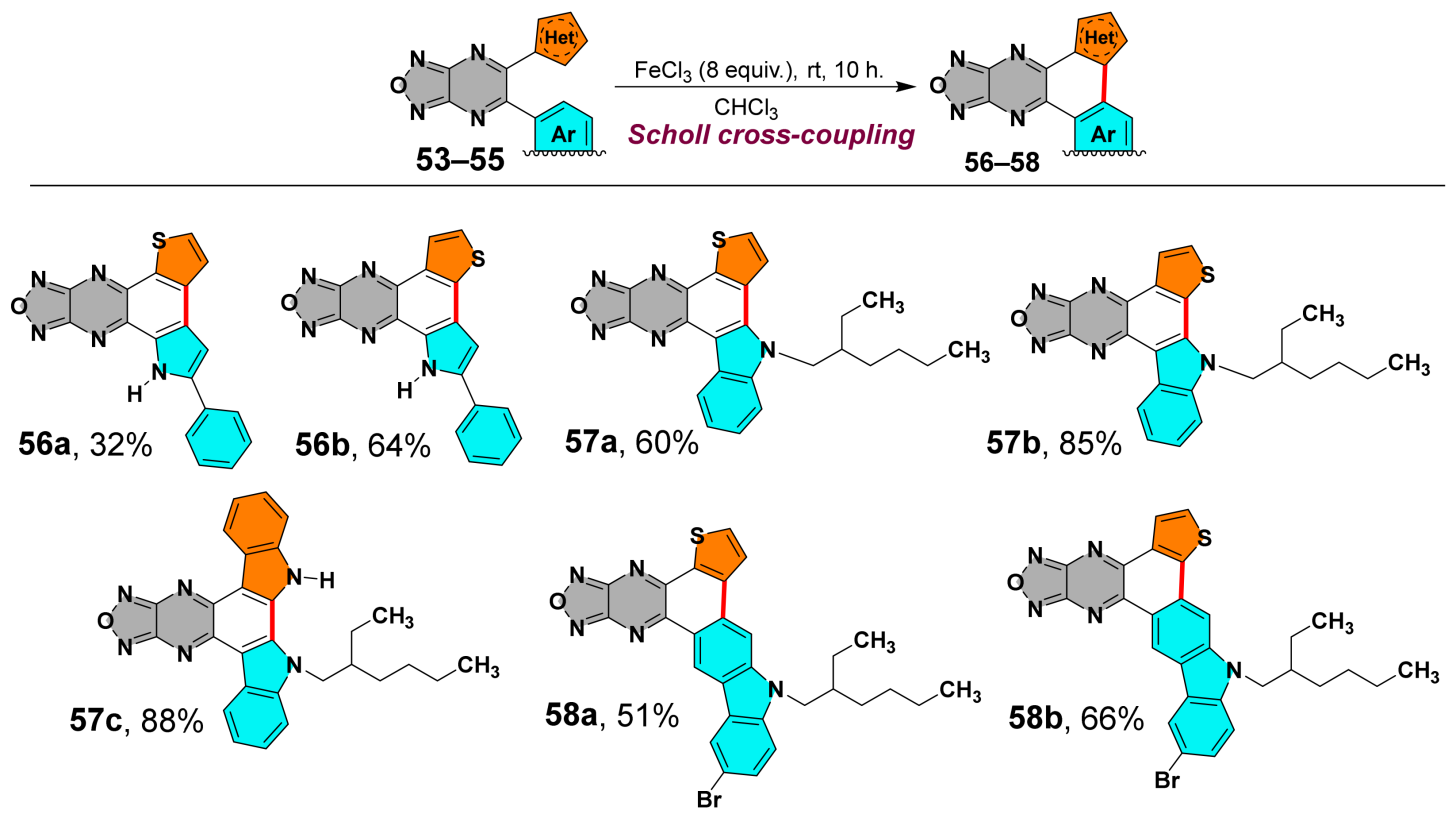
Synthesis of asymmetrical derivatives of [1,2,5]oxadiazolo-fused heteroanalogs of 1,4-diazatriphenylene (**56**–**58**).

This synthetic approach was applied to synthesize more complicated derivatives, including substituted [1,2,5]oxadiazolo[3,4-*b*]pyrrolo[2,3-*f*]thienoquinoxalines (**56a**,**b**), [1,2,5]oxadiazolo[3′,4′:5,6]pyrazino[2,3-*c*]thienocarbazoles (**57a**,**b**), [1,2,5]oxadiazolo[3′,4′:5,6]pyrazino[2,3-c]indolo[2,3-a]carbazole (**57c**), and [1,2,5]oxadiazolo[3′,4′:2,3]thienoquinoxalino[6,5-*b*]carbazoles (**58a**,**b**), achieving yields of 32–88% (Scheme 8) [276].

**Scheme 9:**
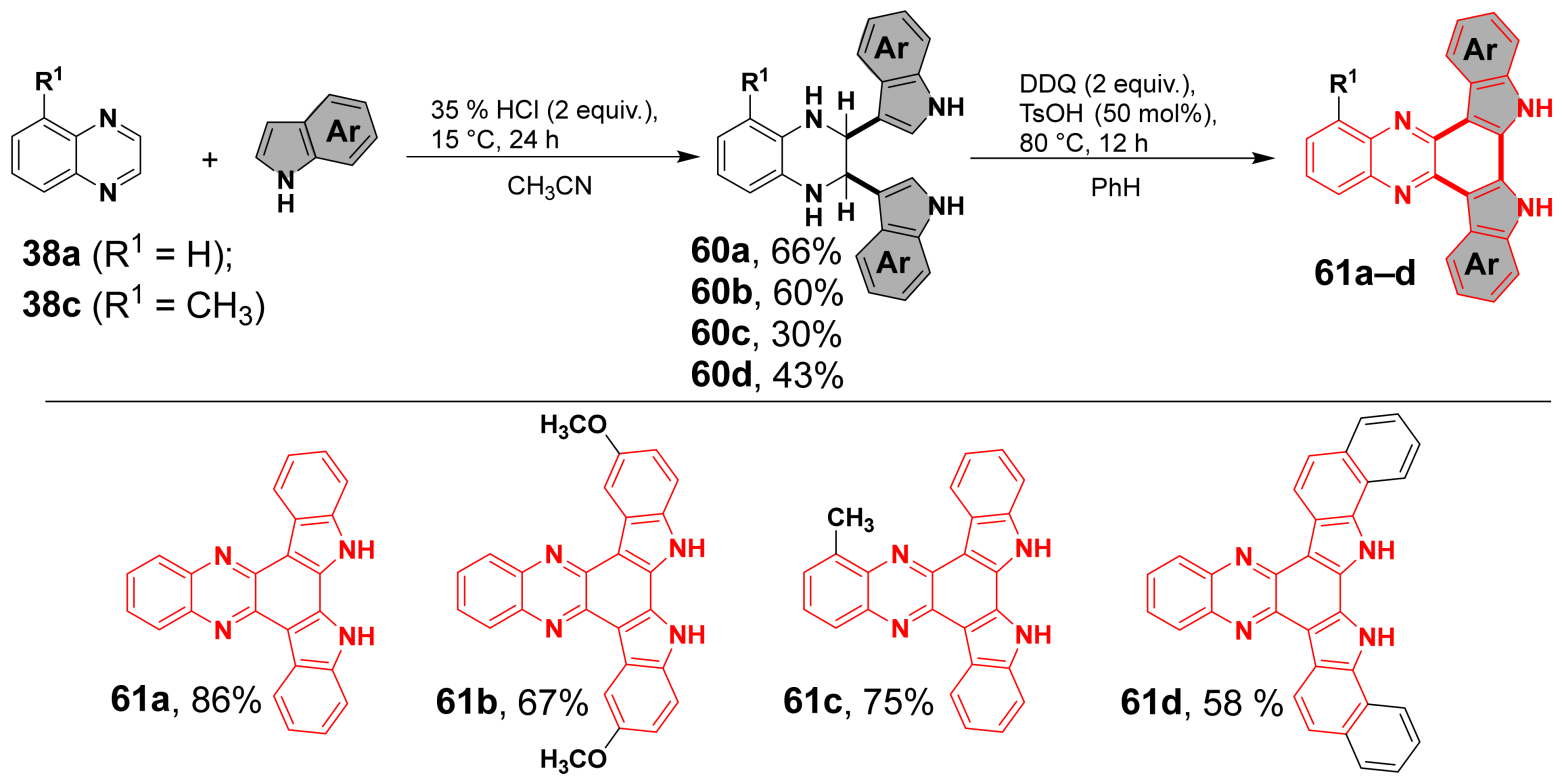
Synthesis of 5,6-dihydrodiindolo[3,2-*a*:2′,3′-*c*]phenazine derivatives (**61a**–**d**).

5,6-Dihydrodiindolo[3,2-*a*:2′,3′-*c*]phenazine derivatives (**61a**–**d**) can be synthesized in a similar manner. The S_N_^H^ reaction between quinoxalines (**38a** and **38c**) and indoles (**59a**–**c**) yields 2,3-substituted 1,4-dihydroquinoxalines (**60a**–**d**), which are subsequently transformed via the Scholl reaction into polycycles **61a**–**d** (Scheme 9) [277].

**Scheme 10:**
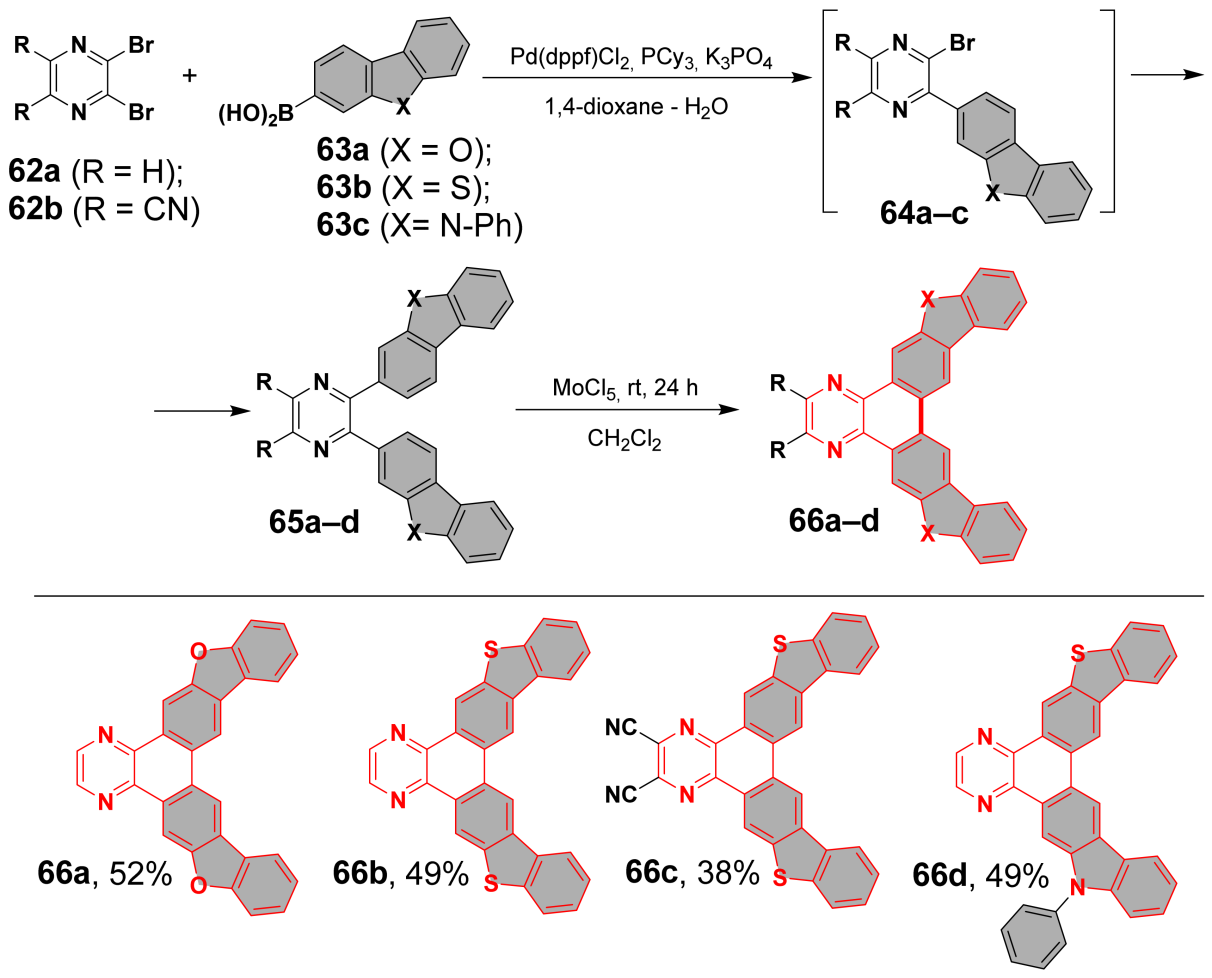
Synthesis of 1,4-diazatriphenylene derivatives (**66a**–**d**).

The Suzuki cross-coupling reaction can also be employed in the syntheses of diarylpyrazines, instead of the S_N_^H^ reactions. This procedure involves the cross-coupling of 2,3-dibromopyrazines (**62a**,**b**) with arylboronic acids (**63a**–**c**), thus affording 2,3-(het)arylpyrazines (**65a**–**d**). These products can be converted further into 1,4-diazatriphenylene derivatives (**66a**–**d**) by using molybdenum(V) chloride, as illustrated in Scheme 10 [278].

## 4. Synthesis Using Photocyclization Reactions

Intramolecular cyclizations of 2,3-di(hetero)aryl-substituted pyrazine derivatives via oxidative photocyclization (the Mallory reaction) represent a convenient, albeit less common, method for the synthesis of heteroanalogs of 1,4-diazatriphenylenes.

**Scheme 11:**
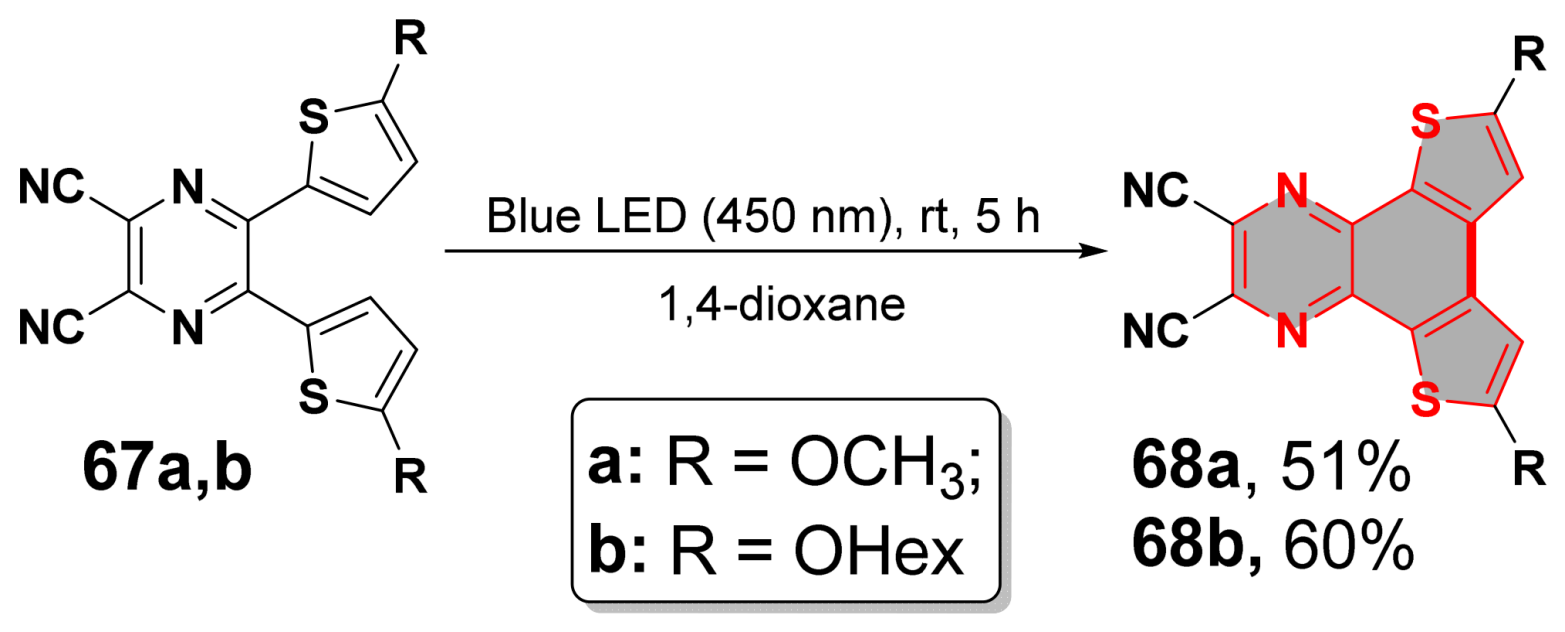
Synthesis of dithieno[2,3-*f*:3′,2′-*h*]quinoxalines (**68a**,**b**).

For example, dithieno[2,3-*f*:3′,2′-*h*]quinoxalines (**68a**,**b**) were readily prepared in yields up to 60% via Royal Blue LED (450 nm) irradiation of 5,6-di(thiophen-2-yl)pyrazine-2,3-dicarbonitriles (**67a**,**b**) (Scheme 11) [279].

**Scheme 12:**
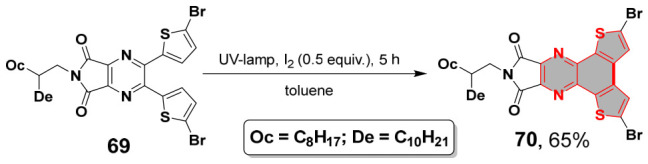
Synthesis of dithieno[2,3-*f*:3′,2′-*h*]quinoxaline derivative (**70**).

**Scheme 13:**
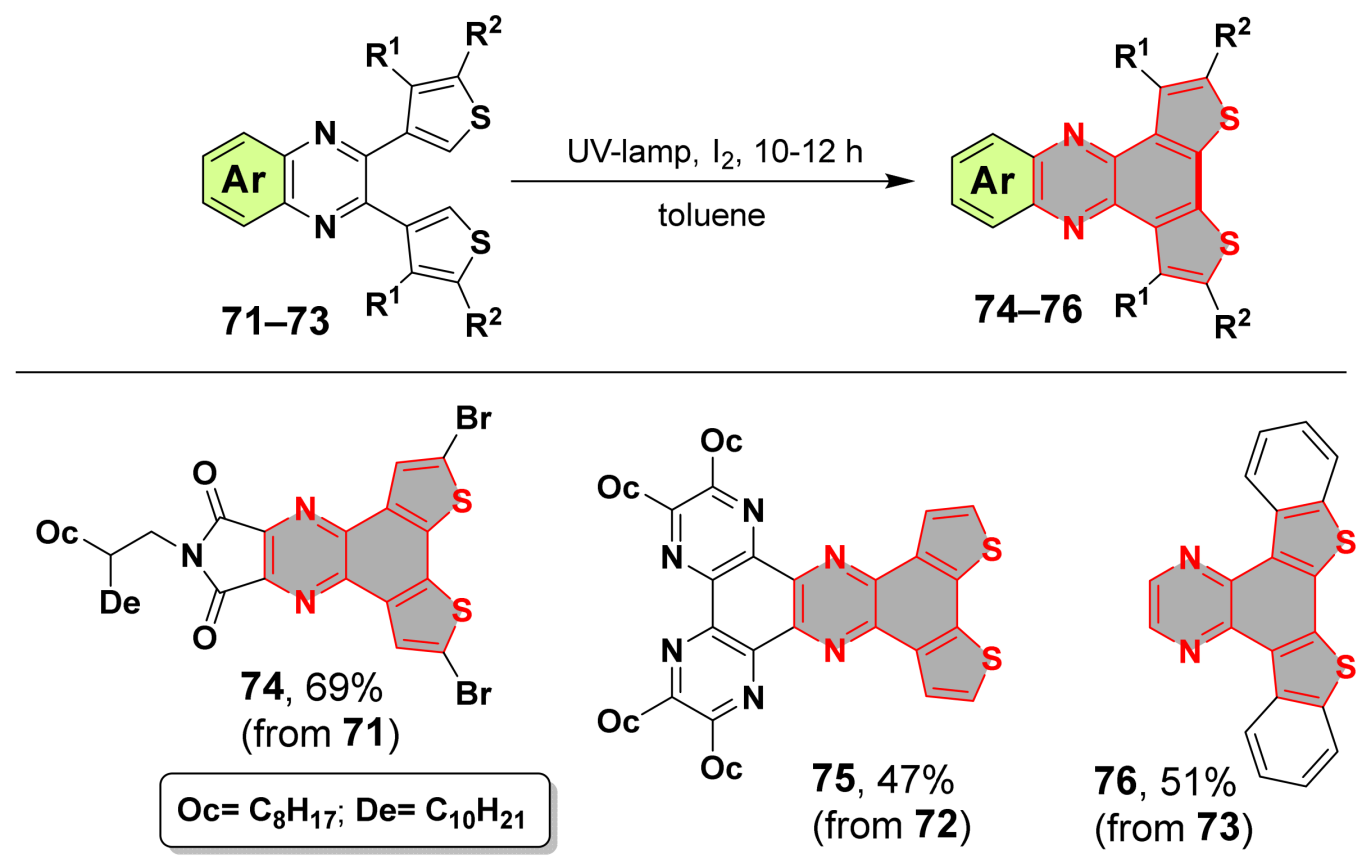
Synthesis of dithieno[3,2-*f*:2′,3′-*h*]quinoxaline derivatives (**74**–**76**).

To enter the Mallory reaction, the compounds 6-R-2,3-di(thiophen-2-yl)-5*H*-pyrrolo[3,4-*b*]pyrazine-5,7(6*H*)-dione (**69**) and various 2,3-di(thiophen-3-yl)pyrazine derivatives (**71**–**73**) were exposed to UV irradiation in the presence of iodine as an oxidizing agent. This oxidative photocyclization resulted in the formation of dithieno[2,3-*f*:3′,2′-*h*]quinoxaline (**70**) and dithieno[3,2-*f*:2′,3′-*h*]quinoxaline (**74**–**76**) derivatives in yields reaching up to 69% (Scheme 12 and Scheme 13) [6,280].

**Scheme 14:**
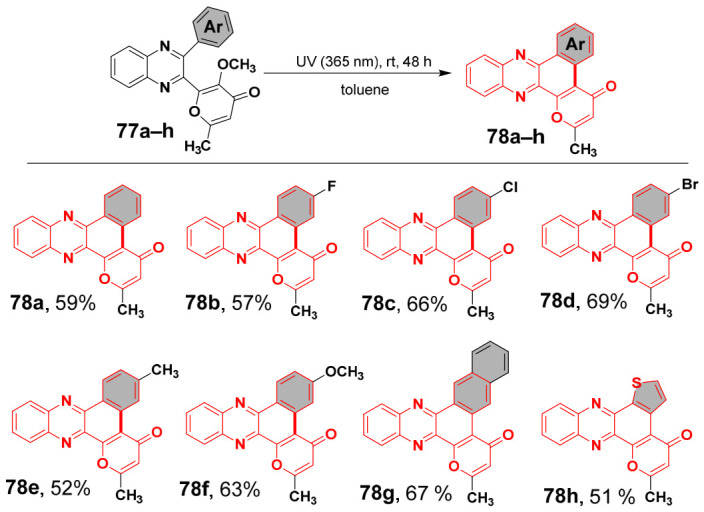
Synthesis of 4*H*-benzo[*a*]pyrano[3,2-*c*]phenazin-4-ones (**78a**–**g**) and 4*H*-pyrano[2,3-*a*]thieno[3,2-*c*]phenazin-4-one (**78h**) derivatives.

Another example of application of photochemical cyclization reactions for the syntheses of 4*H*-benzo[*a*]pyrano[3,2-*c*]phenazin-4-ones (**78a**–**g**) and 4*H*-pyrano[2,3-*a*]thieno[3,2-*c*]phenazin-4-one (**78h**) involves exposing substituted 2-[3-(het)arylquinoxalin-2-yl]-4*H*-pyran-4-ones (**77a**–**h**) to UV radiation (365 nm) for 48 h (Scheme 14) [281].

## 5. Alternative Reactions for the Synthesis of 1,4-Diazatriphenylene and Related Scaffolds

Reports on the construction of 1,4-diazatriphenylene scaffolds and related structures that involve various intermolecular and intramolecular reactions, whether catalyzed by transition metals or not, are significantly less common.

**Scheme 15:**
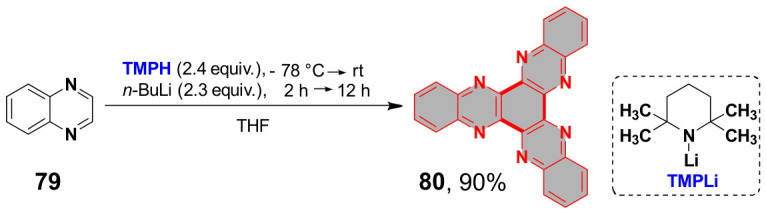
Synthesis of diquinoxalino[2,3-*a*:2′,3′-*c*]phenazine (**80**).

A convenient method for preparing 1,4,5,8,9,12-hexaazatriphenylene (**80**) in 90% yield has been reported in the literature. This method involves the self-realized S_N_^H^ reaction of quinoxaline (**79**), induced by lithiation with **TMPLi**, generated in situ from **TMPH** and *n*-butyllithium. Notably, neither additional additives nor halogen-containing starting materials or transition-metal catalysts were required (Scheme 15) [282].

**Scheme 16:**
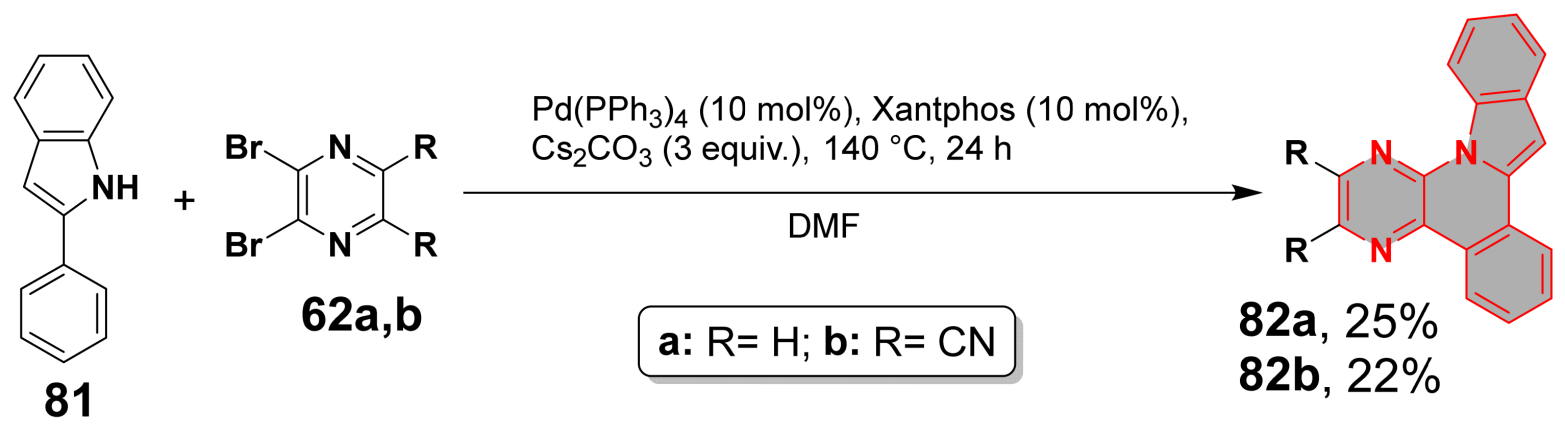
Synthesis of substituted indolo[2,1-*a*]pyrazino[2,3-*c*]isoquinolines (**82a**,**b**).

Another example of the intermolecular cyclization leading to indolo[2,1-*a*]pyrazino[2,3-*c*]isoquinoline derivatives (**82a**,**b**) is the Buchwald–Hartwig cross-coupling between 2-phenyl-1*H*-indole (**81**) and 2,3-dibromopyrazines (**62a**,**b**) (Scheme 16) [283].

In addition to the Scholl reaction, other intramolecular processes can be used to synthesize 1,4-diazatriphenylenes, such as the Cadogan reaction and the intramolecular S_N_^H^ reaction.

**Scheme 17:**
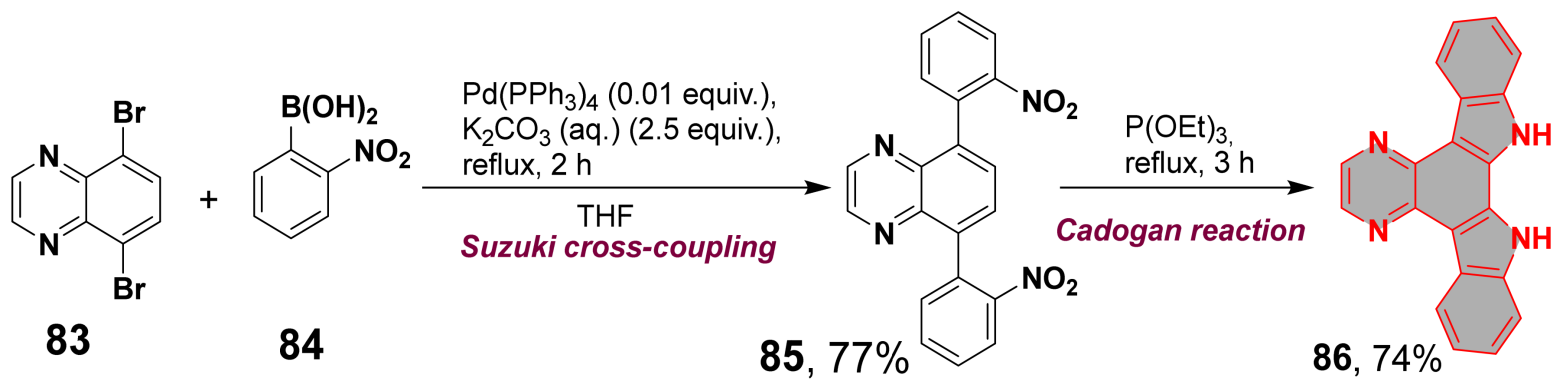
Synthesis of 13,14-dihydroindolo[2,3-*a*]pyrazino[2,3-*c*]carbazole (**86**).

Specifically, the Suzuki cross-coupling reaction between 5,8-dibromoquinoxaline (**83**) and 2-nitrophenylboronic acid (**84**) yields 5,8-*bis*(2-nitrophenyl)quinoxaline (**85**). This compound is subsequently transformed into 13,14-dihydroindolo[2,3-*a*]pyrazino[2,3-*c*]carbazole (**86**) in a high yield under Cadogan reaction conditions (Scheme 17) [284].

**Scheme 18:**
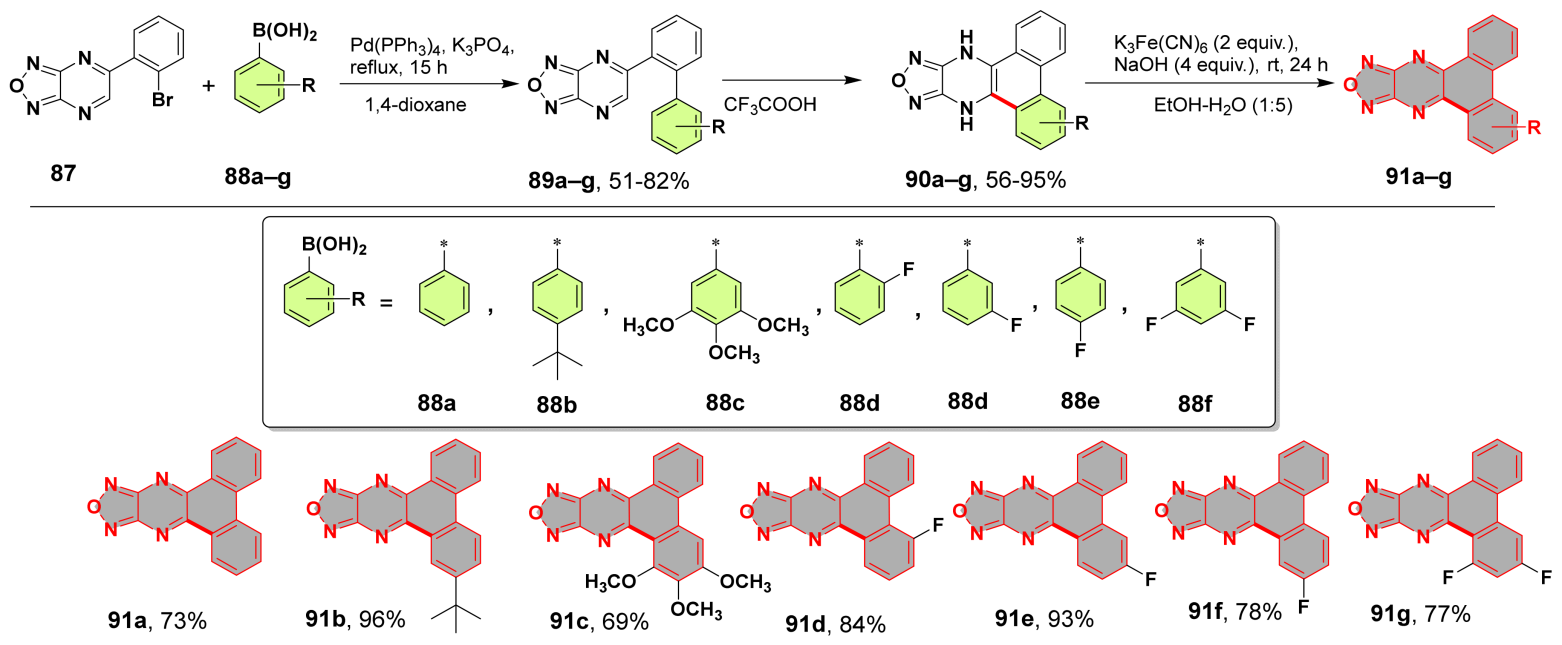
Synthesis of dibenzo[*f*,*h*][1,2,5]oxadiazolo[3,4-*b*]quinoxalines (**91a**–**g**).

A noteworthy example is the Suzuki reaction between 5-(2-bromophenyl)[1,2,5]- oxadiazolo[3,4-b]pyrazine (**87**) and phenylboronic acids (**88a**–**g**), which affords 5-*bis*(aryl)-substituted furazano[3,4-*b*]pyrazines (**89a**–**g**). The latter can undergo the intramolecular S_N_^H^ reaction easily, provided potassium hexacyanoferrate(III) is used to oxidize the intermediate σ^H^-adducts (**90a**–**g**). This procedure yields the desired dibenzo[*f*,*h*][1,2,5]oxadiazolo[3,4-*b*]-quinoxalines (**91a**–**g**) in high yields, reaching up to 96% (Scheme 18) [285].

**Scheme 19:**
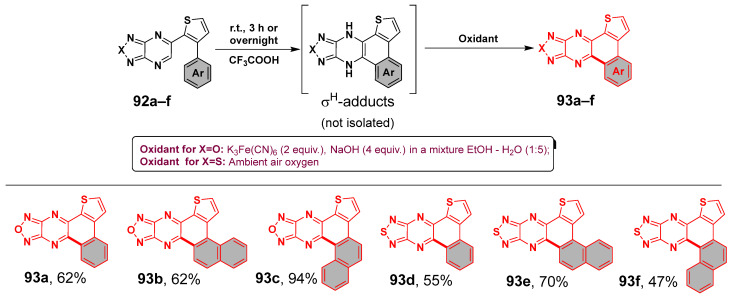
Synthesis of benzo[*f*][1,2,5]chalcogenodiazolo[3,4-*b*]thieno[3,2-*h*]quinoxalines and their benzo-fused derivatives (**93a**–**f**).

The same synthetic method has later been applied to develop a series of new benzo[*f*][1,2,5]chalcogenodiazolo[3,4-*b*]thieno[3,2-*h*]quinoxalines and their benzo-fused derivatives (**93a**–**f**), as shown in Scheme 19 [286].

It is important to highlight that intramolecular S_N_^H^ reactions represent a widely used method for the synthesis of polycyclic systems. This synthetic technique has also been adapted for the preparation of 1,3-diazatriphenylene derivatives and their heteroanalogs [287,288].

**Scheme 20:**
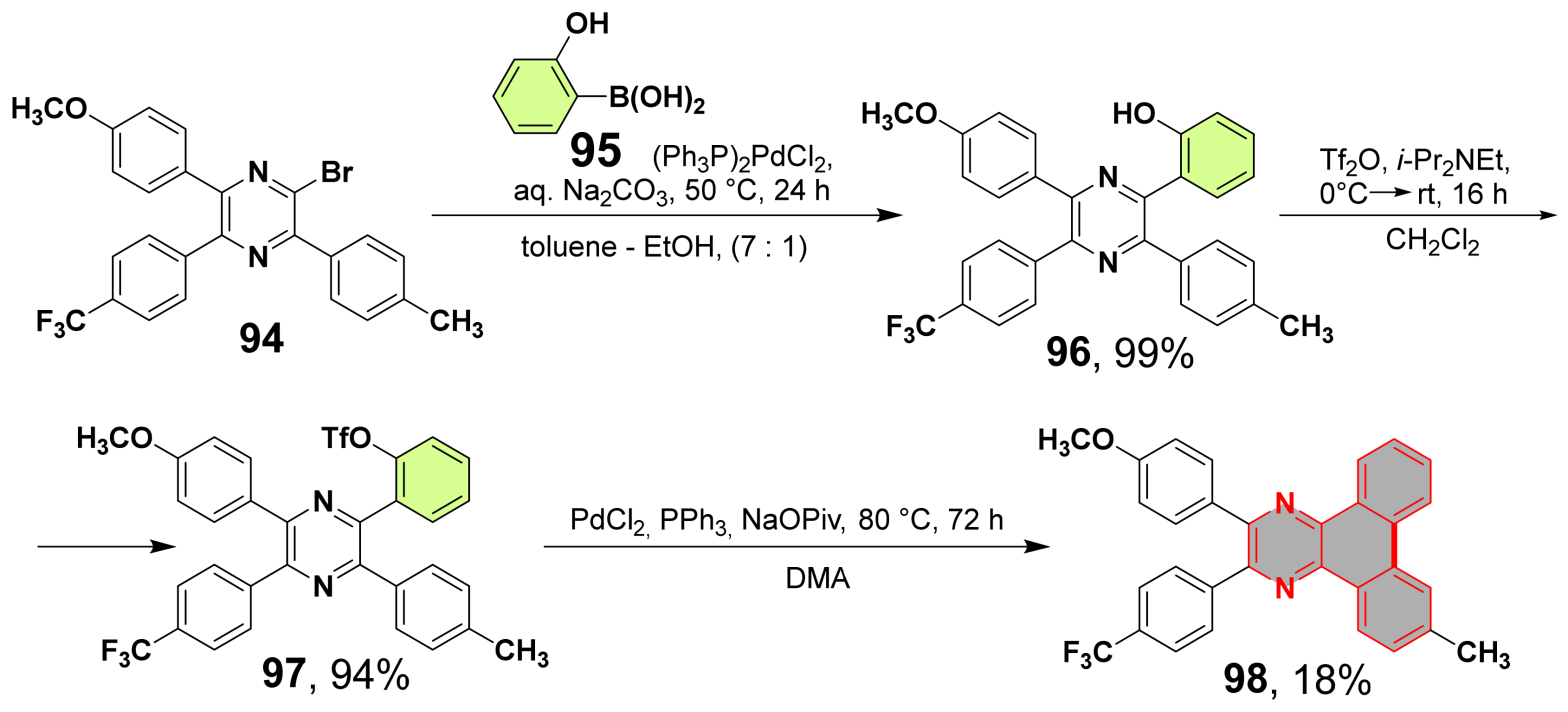
Synthesis of 2-(4-methoxyphenyl)-7-methyl-3-(4-(trifluoromethyl)phenyl)dibenzo[*f*,*h*]quinoxaline (**98**).

Palladium-catalyzed intramolecular cross-coupling reactions and direct C–H bond functionalization are effective tools for assembling 1,4-diazatriphenylenes. For instance, the compound 2,3-Di(aryl)dibenzo[*f*,*h*]quinoxaline (**98**) was prepared through the palladium-catalyzed intramolecular functionalization of the C(*sp*^2^)–H bond in the corresponding 2,3,5,6-tetraphenyl-substituted pyrazine (**97**). This pyrazine was obtained via Suzuki cross-coupling of an appropriate bromo derivative (**94**) (Scheme 20) [289].

**Scheme 21:**
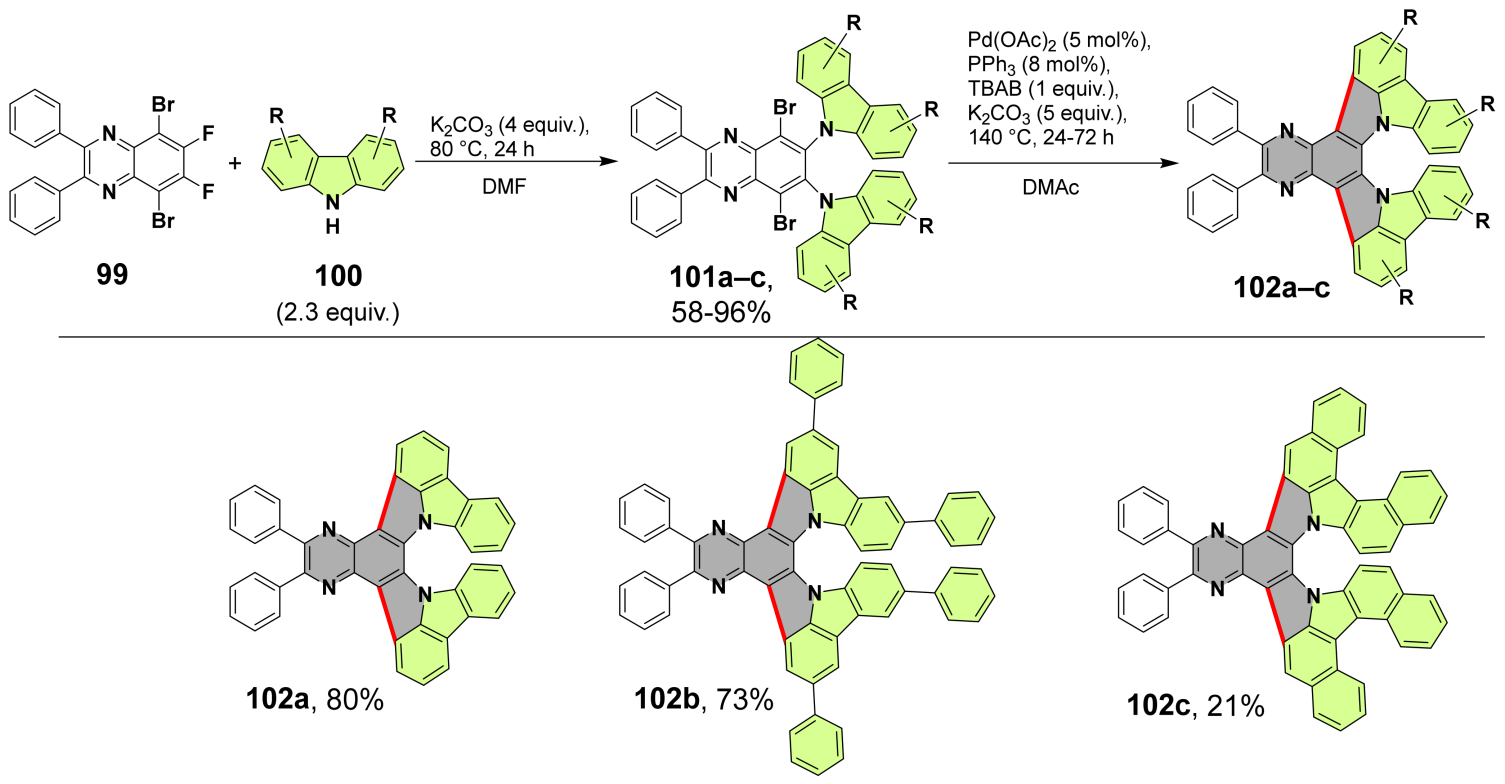
Synthesis of polycyclic systems **102a**–**c**.

In another example, polycyclic systems **102a**–**c** were prepared in moderate yields by palladium-catalyzed intramolecular cyclizations of 9,9′-(5,8-dibromo-2,3-diphenylquinoxaline-6,7-diyl)*bis*(9*H*-carbazole) derivatives (**101a**–**c**). These substances are readily formed through the *ipso*-substitution of bromo atoms in 5,8-dibromo-6,7-difluoro-2,3-diphenylquinoxaline (**99**) with carbazole derivatives (**100a**–**c**), as shown in Scheme 21 [290].

**Scheme 22:**
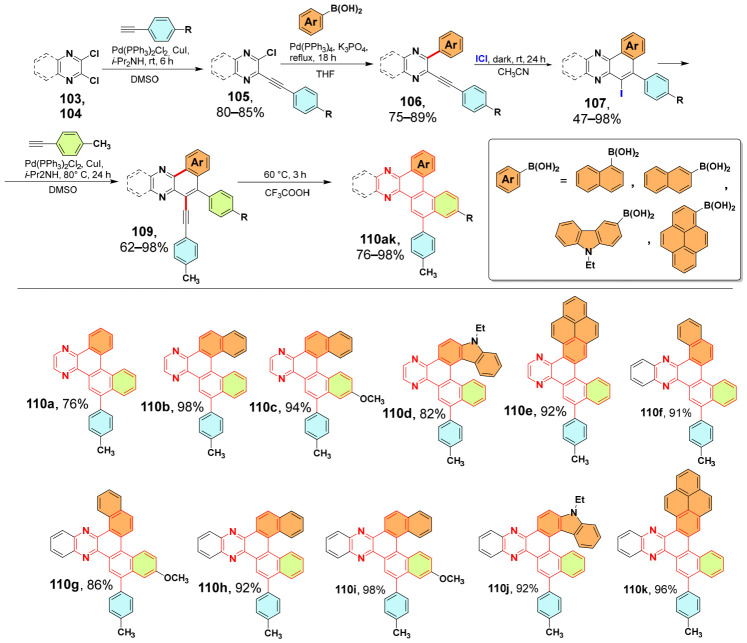
Synthesis of benzo[*f*]naphtho[1,2-*h*]quinoxaline derivatives (**110a**–**k**).

Benzo[*f*]naphtho[1,2-*h*]quinoxaline derivatives (**110a**–**k**), which are [*n*]helicenes (where *n* = 4–6), can be obtained from either 2,3-dichloropyrazine (**103**) or 2,3-dichloroquinoxaline (**104**). This protocol involves a sequence of reactions, including Sonogashira and Suzuki cross-couplings, followed by intramolecular cyclizations proceeding in the presence of iodine monochloride and/or trifluoroacetic acid (Scheme 22) [291,292,293,294].

**Scheme 23:**
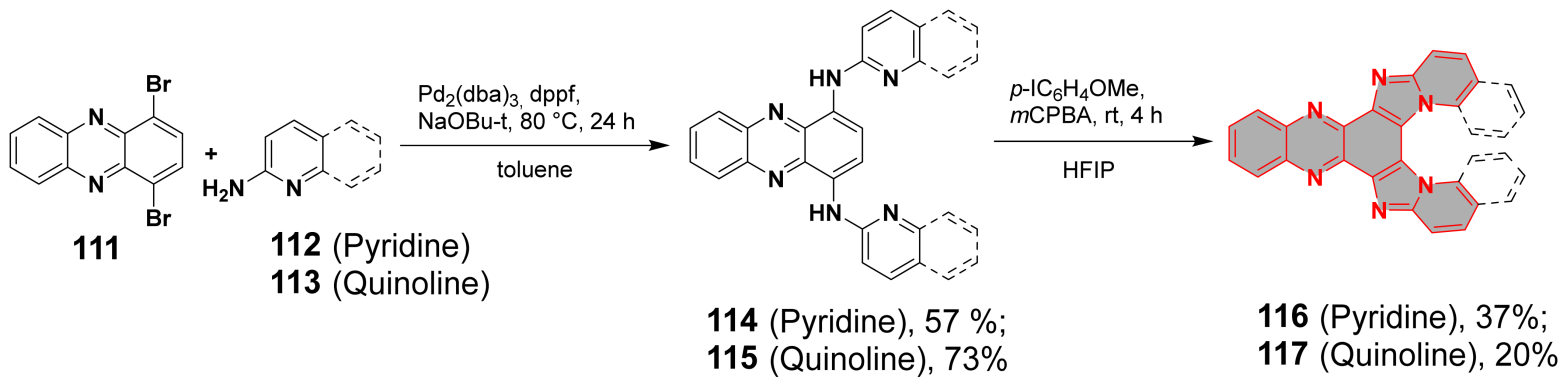
Synthesis of polyaza[5]helicene (**116**) and polyaza[7]helicene (**117**).

Polyaza[5]helicene (**116**) and polyaza[7]helicene (**117**) can be readily prepared through the Buchwald–Hartwig reaction between 1,4-dibromophenanzine (**111**) and 2-aminopyridine (**112**) [or 2-aminoquinoline (**113**)], followed by intramolecular oxidative N–H/C–H coupling, thus leading to 1,4-di(arylamino)phenazines (**162** and **163**) (Scheme 23) [295].

**Scheme 24:**
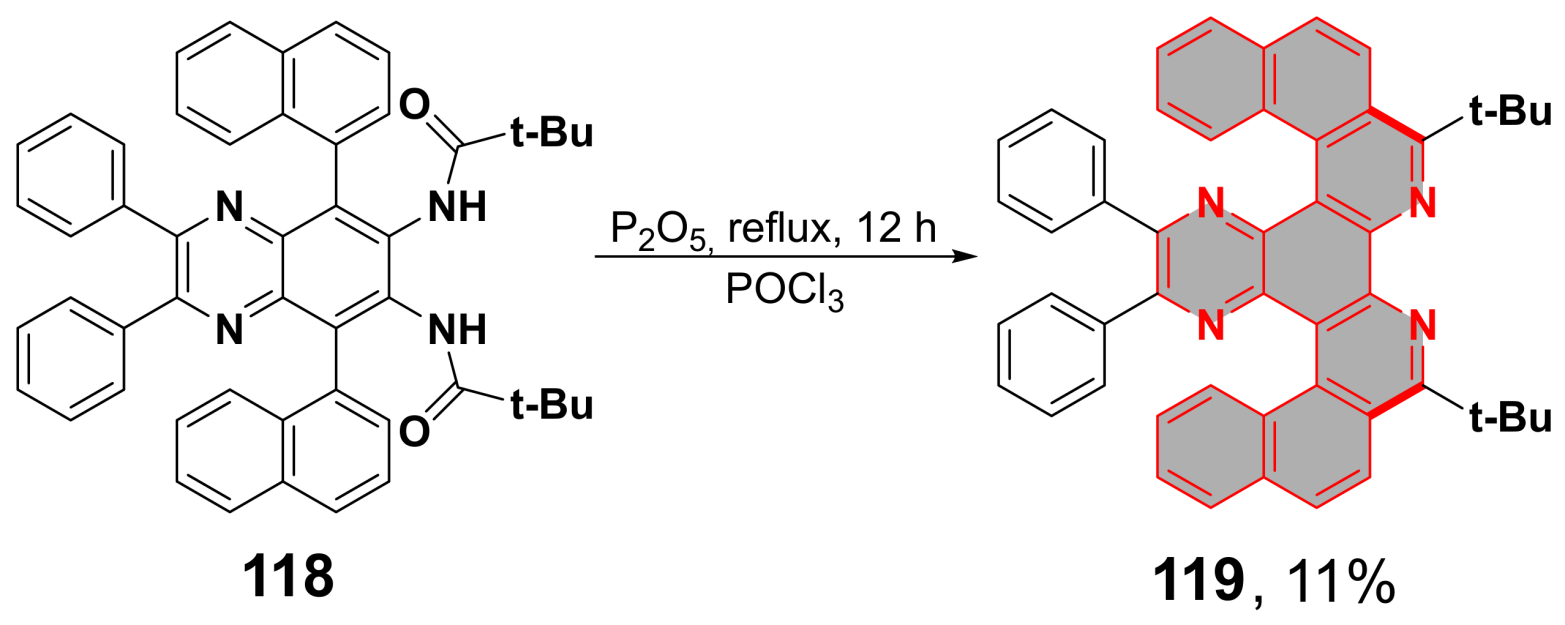
Synthesis of 7,10-di-*tert*-butyl-18,19-diphenyldinaphtho[2,1-*c*:1′,2′-*i*]pyrazino[2,3-*f*][1,10]phenanthroline (**119**).

It is worth noting that aza[5]helicene derivatives can be synthesized from polysubstituted quinoxalines through the Bischler–Napieralski reaction. For instance, this method was used to obtain 7,10-di-*tert*-butyl-18,19-diphenyldinaphtho[2,1-*c*:1′,2′-*i*]pyrazino[2,3-*f*][1,10]phenanthroline (**119**) in a moderate yield from 2,3,5,6,7,8-hexasubstituted quinoxaline (**118**) (Scheme 24) [296].

**Scheme 25:**
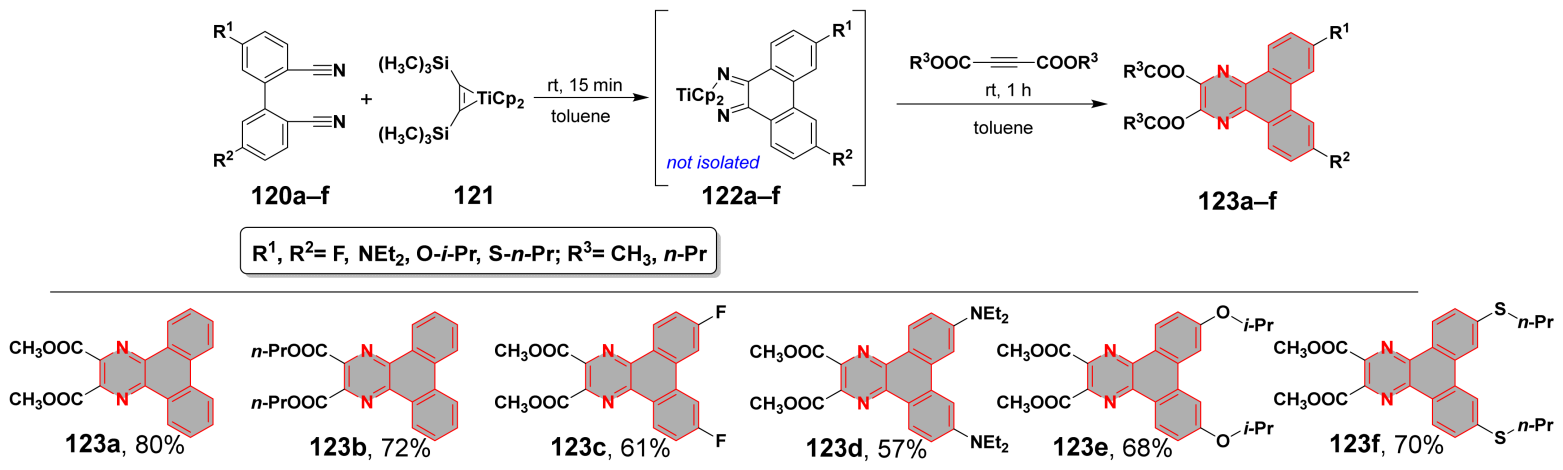
Synthesis of 2,3,7,10-*tetra*-substituted dibenzo[*f*,*h*]quinoxalines (**123a**–**f**).

An interesting method for preparing 2,3,7,10-*tetra*-substituted dibenzo[*f*,*h*]quinoxalines (**123a**–**f**) involves the reductive cyclization of substituted [1,1′-biphenyl]-2,2′-dicarbonitriles (**120a**–**f**), mediated by titanocene (**121**). The resulting intermediate polycyclic aromatic compounds contain a di(aza)titanium cyclopentadiene fragment (**122a**–**f**) and undergo a divergent titanocene transfer reaction to yield 1,4-diazatriphenylenes (**123a**–**f**). The authors have tried to rationalize this process as a formal [2 + 2 + 2]-reaction, leading to the formation of the pyrazine ring (Scheme 25) [297].

**Scheme 26:**
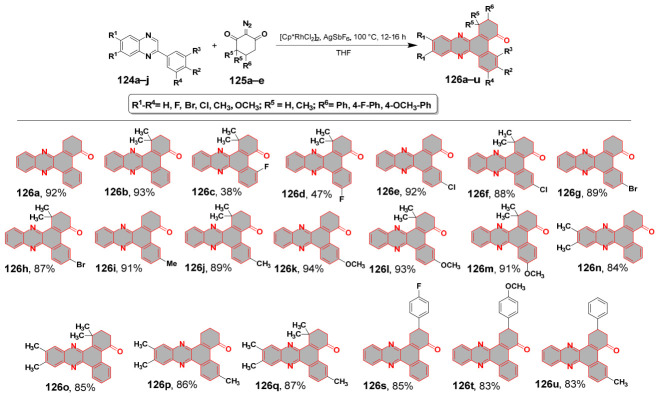
Synthesis of 2,3-dihydrodibenzo[*a*,*c*]phenazin-4(1*H*)-one derivatives (**126a**–**u**).

Another example involves the Rh(III)-catalyzed oxidative annulation of 2-arylquinoxalines (**124a**–**j**) by the action of cyclic 2-diazo-1,3-diketones (**125a**–**e**) through C−H bond activation using AgSbF_6_ as a cocatalyst. This process leads to the formation of 2,3-dihydrodibenzo[*a*,*c*]phenazin-4(1*H*)-ones (**126a**–**u**) in high yields, achieving 94% (Scheme 26) [298].

**Scheme 27:**
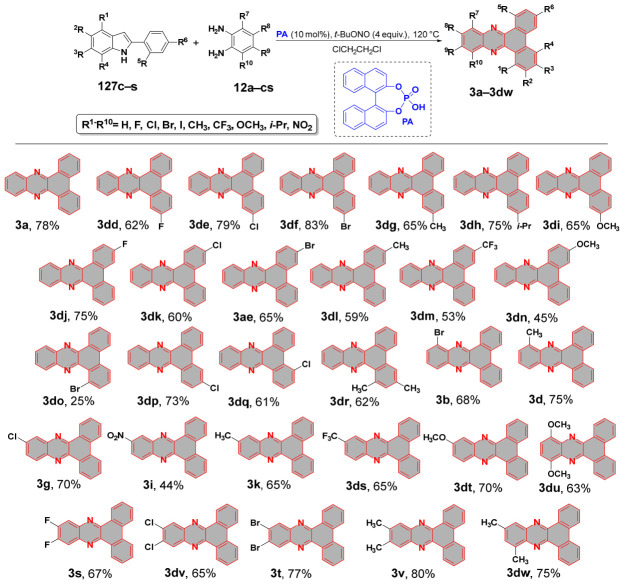
Synthesis of substituted dibenzo[*a*,*c*]phenazines (**3a-wd**).

A notable method for preparing a variety of substituted dibenzo[*a*,*c*]phenazines (**3a-wd**) involves Brønsted acid-catalyzed “skeletal editing” of 2-arylindoles (**127c-s**) using 1,2-diaminoarenes (**12a-cs**) in the presence of *tert*-butyl nitrite (Scheme 27) [299]. The authors have demonstrated that this “skeletal editing” process consists of a *one*-*pot* sequence that includes five reactions: nitrosation, condensation, cyclization, diazotization, and intramolecular electrophilic substitution. However, the authors do not provide details concerning the mechanism of this process.

## 6. Applications of 1,4-Diazatriphenylene Derivatives and Their Heteroanalogs

**Figure 3:**
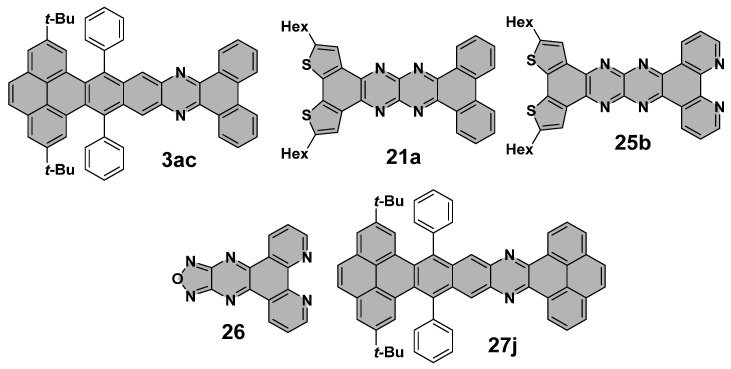
Selected examples of compounds (**3ac**, **21a**, **25b**, **26**, and **27j**) that are promising for utilization in organic field-effect transistors, OLEDs, and solar cells.

Fused 1,4-diazines are well-established and promising components for photo- and/or electroactive materials used in various fields of applications [300]. Derivatives of 1,4-diazatriphenylene and its heteroanalogs (such as compounds **3ac**, **21a**, **25b**, **26**, and **27j**) are promising candidates for developing semiconductor materials for organic electronics (Figure 3). These compounds can be utilized in organic field-effect transistors, light-emitting diodes (OLEDs), and solar cells [75,76,166,219].

**Figure 4:**
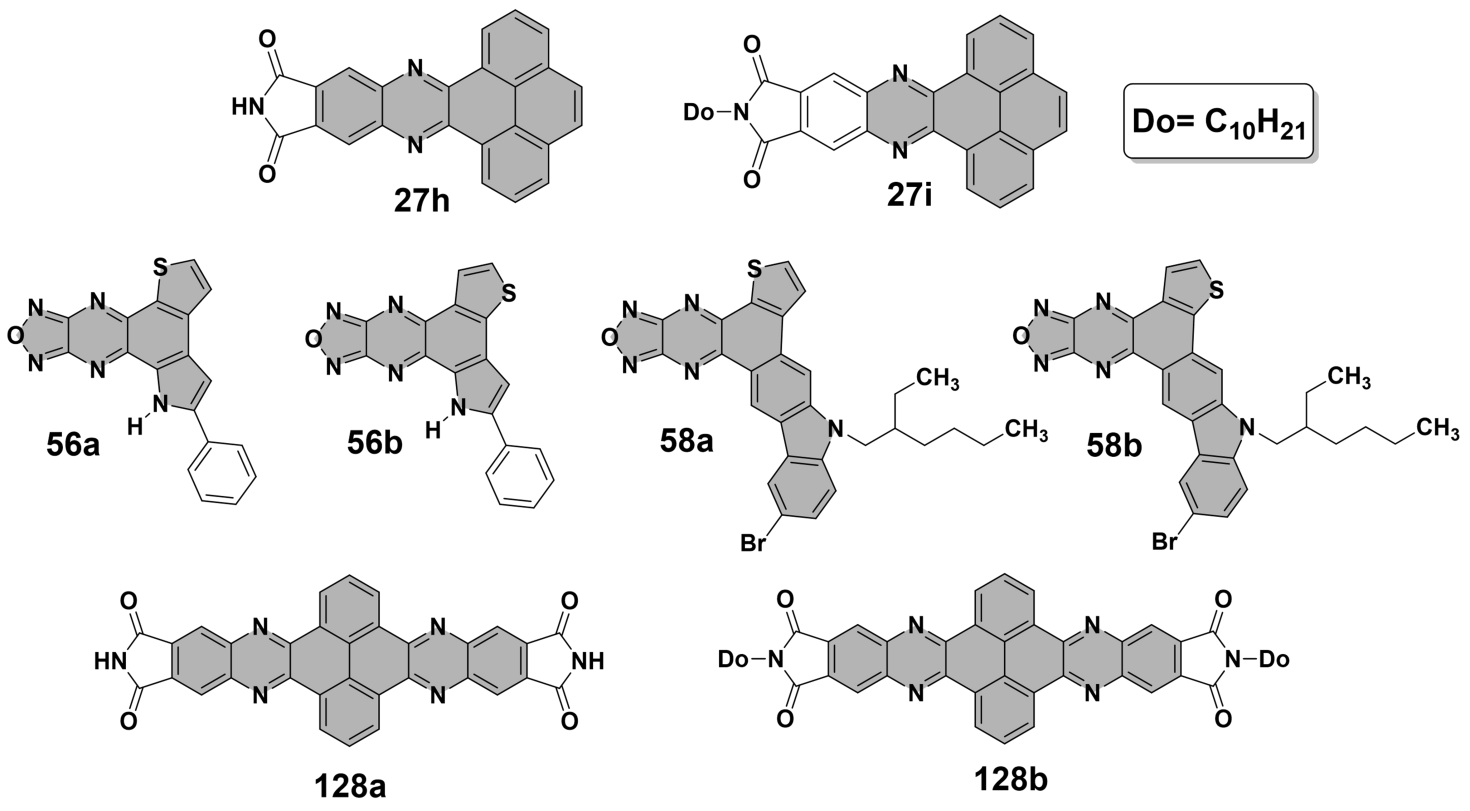
Examples of narrow-gap *n*-type semiconductors based on 1,4-diazatriphenylene and its hetero-fused analogs (**27h**, **27i**, **56a**,**b**, **58a**,**b**, and **128a**,**b**).

In particular, compounds **27h**, **27i**, **56a**,**b**, **58a**,**b**, and **128a**,**b** are considered to be promising as narrow-gap *n*-type semiconductors (Figure 4) [176,193,276]. Electrochemical and electrical conductivity measurements indicate that compound **128a** functions effectively as an *n*-channel semiconductor, demonstrating the highest charge carrier mobility in this series, reaching up to 4.1 cm^2^·V^−1^·s^−1^ [176].

Polycyclic systems incorporating a 1,4-diazatriphenylene scaffold have often been applied as dopants in the emitter layer of thermally activated delayed fluorescence organic light-emitting diodes [18,29,35,48,49,52,57,61,66,67,78,79,80,81,83,84,85,86,93,94,101,102,103,104,105,106,113,114,115,150,156,168,179,190,214,265].

On the other hand, hetero-fused analogs of 1,4-diazatriphenylene have been utilized as non-fullerene acceptors in efficient organic solar cells and as components of polymer solar cells [183,202,206]. Table 3 provides examples of polycycles (**129**–**134**) used in the assembly of high-efficiency solar cells, along with their characteristics.

**Figure 5:**
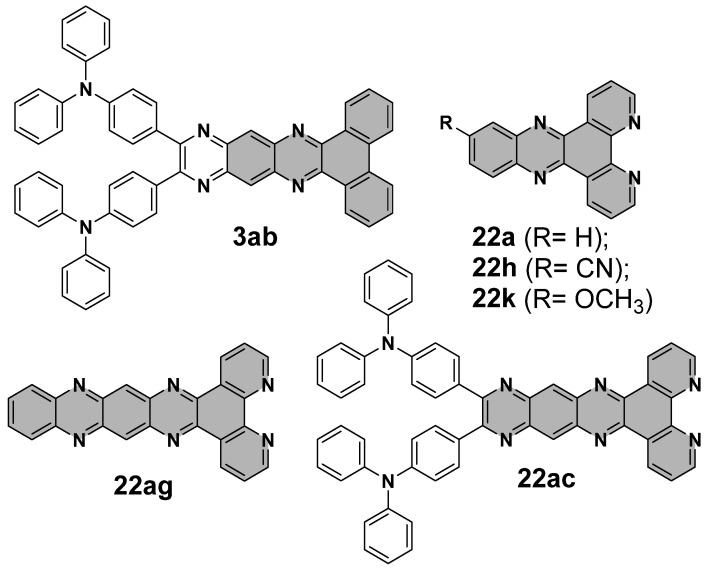
Examples of polycyclic compounds (**3ab**, **22a**, **22h**, **22k**, **22ac**, and **22ag**) utilized as electrochromic materials and components for electrodes in metal-ion batteries.

In addition, some polycyclic compounds have been identified as electrochromic materials (e.g., compounds **3ab**, **22a**, **22h**, **22k**, and **22ac**) (Figure 5) [74,127] and as cathode materials in metal-ion batteries (compounds **22h** and **22ag**) [141,165,229,230,233,235,236,241,245,246,249,250,251,252,253,254].

**Figure 6:**
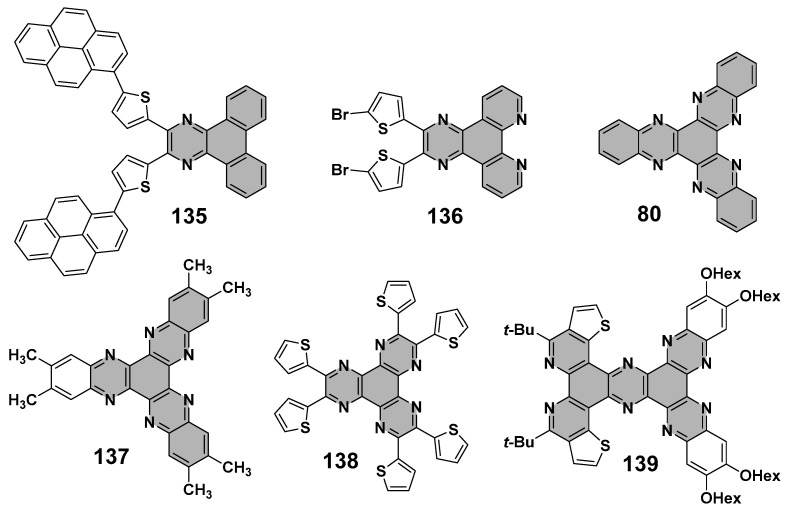
Examples of polycyclic compounds utilized as sensors for detecting nitro-explosives (compound **135**) and metal cations (compounds **80**, **136**–**139**).

**Figure 7:**
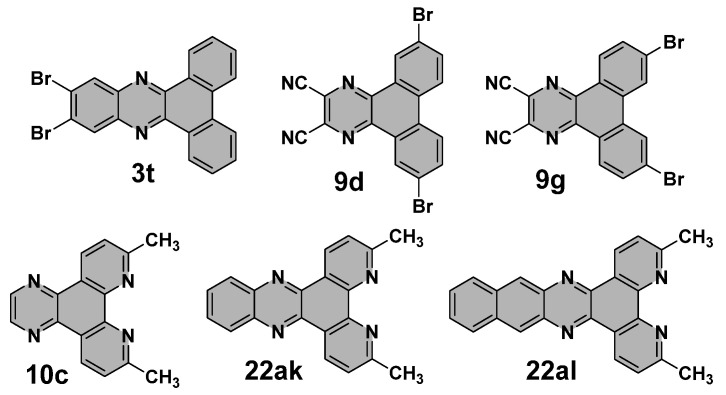
Examples of polycyclic compounds utilized as photocatalysts for various chemical reactions (compounds **3t**, **9d**, **9g**, **10c**, **22ak**, and **22al**).

Additionally, compounds of similar structures are regarded as promising as sensors for detecting nitro-explosives (compound **135**) [211] and metal cations (compounds **80**, **136**–**139**) (Figure 6) [203,212,234,260]. They can also serve as excellent photocatalysts for various chemical reactions (compounds **3t**, **9d**, **9g**, **10c**, **22ak**, and **22al**) (Figure 7) [13,15,27,63,279].

**Figure 8:**
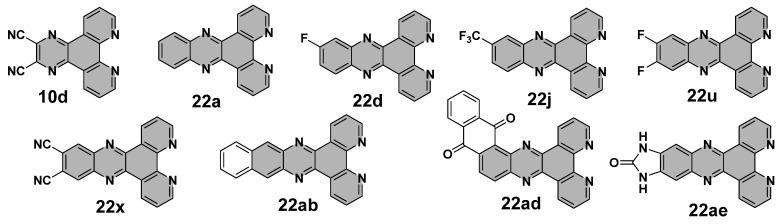
Examples of polycyclic ligands (**10d**, **22a**, **22d**, **22j**, **22u**, **22x**, **22ab**, **22ad**, and **22ae**) used for the synthesis of biologically active metal complexes.

It is important to note that metal complexes of some pyrazino[2,3-*f*][1,10]phenanthroline (**10d**) and dipyrido[3,2-*a*:2′,3′-*c*]phenazine derivatives (**22a**, **22d**, **22j**, **22u**, **22x**, **22ab**, **22ad**, and **22ae**) exhibit a variety of biological activities, such as anticancer [28,124,126,134,149,152,153,161,163,169], antibacterial (compounds **22a**, **22d**, **22j**, **22u**, and **22x**) [123,126,153], and antifungal properties (compound **22a**) (Figure 8) [125].

**Figure 9:**
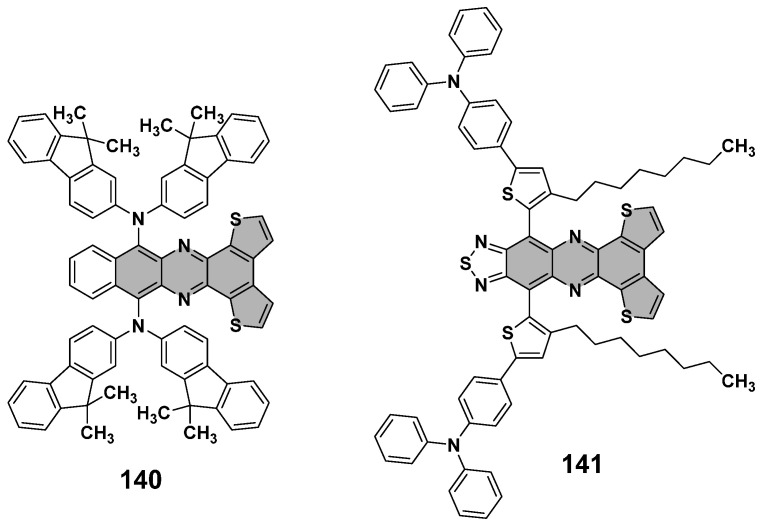
Examples of polycyclic compounds (**140** and **141**) utilized as fluorophores for in vivo bioimaging in the second near-infrared window.

Dithieno[2,3-*a*:3′,2′-*c*]phenazine derivatives (for example, compounds **140** and **141**) are remarkable due to their potential as fluorescence bioimaging agents in the second near-infrared window (1000–1700 nm), which can be utilized for studying cerebrovascular functions (Figure 9) [301,302].

## 7. Conclusions

In this review article, we have attempted to present the latest literature data on the advances in the chemistry of 1,4-diazatriphenylene and its hetero-fused analogs, focusing on various synthetic methodologies, including condensation reactions and intramolecular cyclizations of 2,3-di(hetero)aryl-substituted pyrazine derivatives. These methods incorporate various processes such as oxidative photocyclization (the Mallory reaction), intramolecular cyclodehydrogenations proceeding through the Scholl reaction, or nucleophilic aromatic substitutions of hydrogen (the S_N_^H^ reactions) in the series of 2-*bis*(hetero)aryl-substituted 1,4-diazine derivatives.

Additionally, the review examines the potential applications of these compounds as fluorescent and/or semiconducting materials, as well as their roles in coordination chemistry and biological properties. This review covers the literature from 2018 to March 2026, complementing the data discussed in our previous publication [4].

As follows from the review, the main method for the synthesis of 1,4-diazatriphenylenes and their condensed heteroanalogs is based on condensation of previously obtained 1,2-diamines with 1,2-diketones, and organic electronics, namely charge-transport and light-emitting materials for OLEDs, field-effect transistors, and solar cells, appears to be the main area of their potential applications. Recent publications [10,11] indicate that one of the great challenges in the chemistry of 1,4-diazines and their fused derivatives is the development of synthetic methods based on direct C–H bond functionalization.

We believe that S_N_^H^ reactions, which offer innovative approaches to sustainable and green chemistry for modifying polycyclic scaffolds, have often been underestimated, resulting in their synthetic potential not being fully utilized. Additionally, [1,2,5]oxadiazolo-annulated polycyclic systems are recognized as versatile building blocks for synthesizing a range of 1,4-diazatriphenylene derivatives, thereby significantly expanding their synthetic possibilities. This versatility primarily stems from the ability of the furazan ring to be readily reduced to yield the corresponding 1,2-diamino compounds. Furthermore, these derivatives can be transformed not only into condensed imidazoles but also into a variety of 1,2,5-chalcogenadiazole derivatives [303,304]. The authors believe this review will be of help to organic chemists in developing promising materials related to polycyclic (hetero)aromatics and their metal complexes.

## Figures and Tables

**Table 1:** Selected examples of the preparation of derivatives of dibenzo[*a*,*c*]phenazine (**9**), pyrazino[2,3-*f*][1,10]phenatroline (**10**), and phenanthro[4,5-*fgh*]quinoxaline (**11**).

Entry	1,2-Diketones (4–6)	1,2-Diamines (7 or 8)	Conditions	Products (9–11) —Isolated Yield	Ref.
1	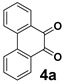	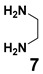	**Fe_3_O_4_@EDA-SO_3_H**, MNR catalyst, rt, 15 min/EtOH 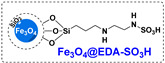	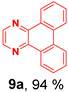	[12]
2	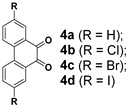	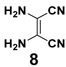	reflux, 12 h/AcOH	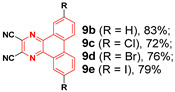	[13]
3	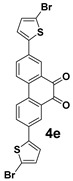	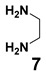	40 °C, 8 h/solvent-free	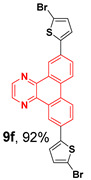	[14]
4	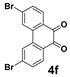	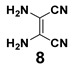	reflux, 12 h/AcOH	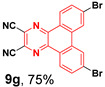	[13]
5	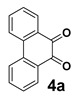	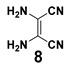	reflux, 12 h/AcOH	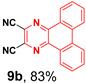	[15]
6		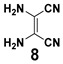	*nano*-Ca(IO_3_)_2_, rt, 9 min/AcOH	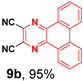	[16]
7	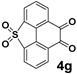	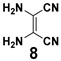	110 °C, 2 h/AcOH	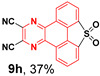	[17]
8	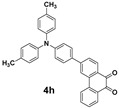	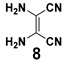	36% HCl, 40 °C, 48 h/EtOH–THF (1:1)	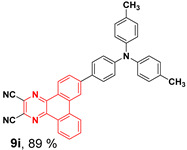	[18]
9	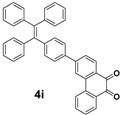	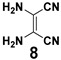	reflux, 6 h/AcOH	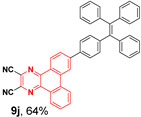	[19]
10	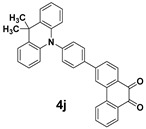	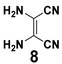	36% HCl, 40 °C, 48 h/EtOH–THF (1:1)	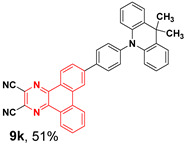	[18]
11	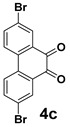	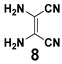	1. TsOH (cat.), reflux, 5 h/EtOH 2. reflux, 12 h/AcOH	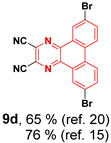	[15,20]
12	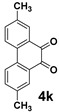	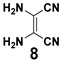	60 °C, 24 h/AcOH	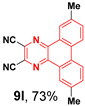	[21]
13	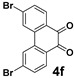	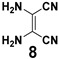	1. TsOH (cat.), reflux, 5 h/EtOH 2. reflux, 12 h/AcOH 3. reflux, 3 h/AcOH	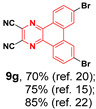	[15,20,22]
14	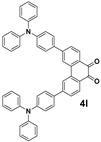	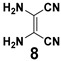	125 °C, 12 h/AcOH	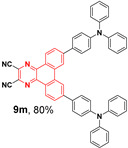	[23]
15	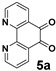	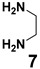	60 °C, 12 h/H_2_O	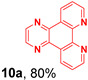	[24,25]
16	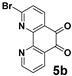	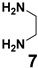	reflux, 4 h/THF	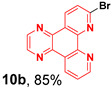	[26]
17	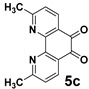	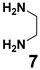	TsOH (cat.), reflux, 16 h/EtOH	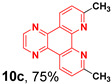	[27]
18	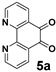	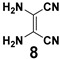	reflux, 4 h/EtOH	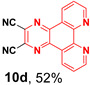	[28]
19	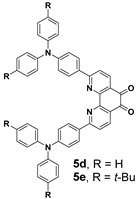	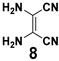	For **R** = **H**: 1. reflux, 12 h/AcOH 2. 120 °C, 12 h/AcOH For **R** = ***t*-Bu**: 120 °C, 20 h/AcOH	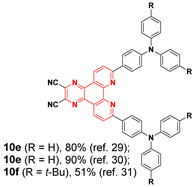	[29,30,31]
20	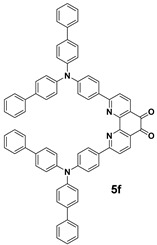	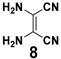	reflux, 12 h/AcOH	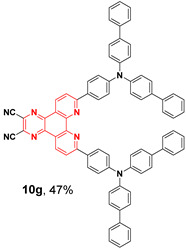	[29]
21	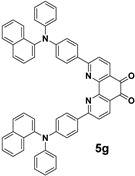	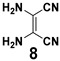	reflux, 12 h/AcOH	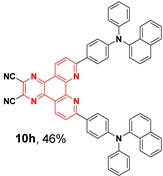	[29]
22	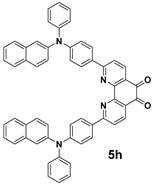	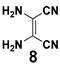	reflux, 12 h/AcOH	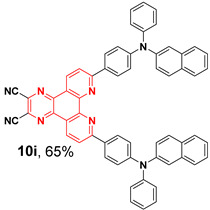	[29]
23	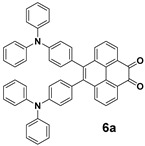	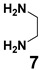	reflux, 12 h/AcOH–EtOH (1:1)	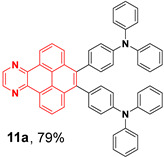	[32]
24	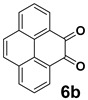	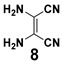	1. reflux, 24 h/AcOH 2. 80 °C, 19 h/AcOH–EtOH (1:1)	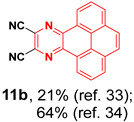	[33,34]
25	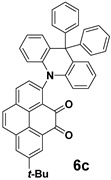	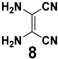	reflux, 8 h/AcOH	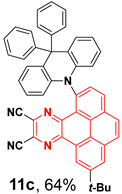	[35]
26	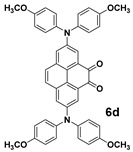	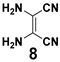	80 °C, 15 h/AcOH–EtOH (1:1)	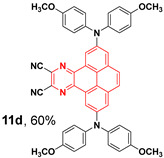	[36]
27	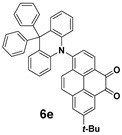	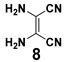	reflux, 8 h/AcOH	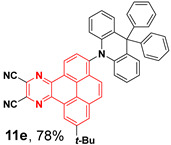	[35]
28	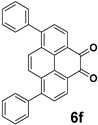	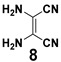	reflux, 24 h/AcOH	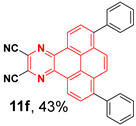	[33]

**Table 2:** Synthesis of 1,4-diazatriphenylene derivatives (**3** and **18**–**21**), pyrazino[2,3-*f*][1,10]phenatrolines (**22**–**26**), and phenanthro[4,5-*fgh*]quinoxalines (**27**, **28**).

Entry	1,2-Diketones (4–6)	1,2-Diamines (12–17)	Conditions	Products (3, 18–28) —Isolated Yield	Ref.
1	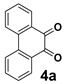	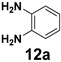	1. *nano*-*γ*-Fe_2_O_3_-SO_3_H, 120 °C, 1 h/solvent-free 2. **Fe_3_O_4_@APTES@isatin-SO_3_H**, MNR catalyst, rt, 20 min/EtOH 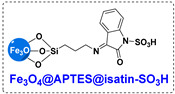 3. **Ionic liquid [BBSA-DBN][HSO_4_]** (3 mol%), 80 °C, 35 min/EtOH 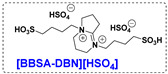 4. amorphous Fe nanoparticles, rt, 7 min/H_2_O 5. SiO_2_/**[SEP]Cl**, 65 °C, 2 h/EtOH 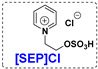 6. HOOCCH_2_C(=CH_2_)COOH, rt, 1 h/H_2_O 7. **CSA** (20 mol%), rt, 1 h/EtOH–H_2_O (1:1) 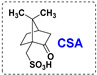 8. MW, 20–22 bar, 230 °C, 10 min/5% AcOH 9. *nano*-Ca(IO_3_)_2_, rt, 3 min/AcOH 10. mesoporous **TG-PSSA-co-ACA**, rt, 5 min/H_2_O 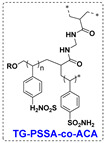 11. **Fe_3_O_4_@EDA-SO_3_H**, MNR catalyst, rt, immediately/EtOH 12. reflux, 24 h/EtOH 13. **[DABCO](SO_3_H)_2_CoCl_4_**, reflux, 18 min/H_2_O 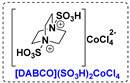	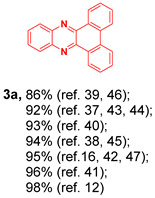	[12,16,37,38,39,40,41,42,43,44,45,46,47]
2	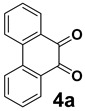	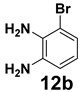	1. reflux, 8 h/AcOH 2. 110 °C, 12 h/AcOH	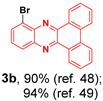	[48,49]
3		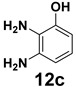	reflux, 2 h/AcOH–EtOH (1:1)	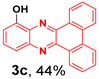	[50]
4		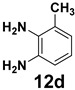	**CSA** (20 mol%), rt, 1 h/EtOH–H_2_O (1:1)	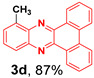	[43]
5		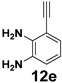	75 °C, 18 h/EtOH–CH_2_Cl_2_ (5:1)	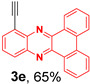	[51]
6		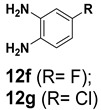	HOOCCH_2_C(=CH_2_)COOH, rt, 1 h/H_2_O	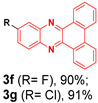	[42]
7		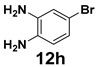	1. reflux, 8 h/AcOH 2. reflux, 8 h/AcOH 3. SiO_2_/**[SEP]Cl**, 65 °C, 1 h 45 min/EtOH 4. 110 °C, 12 h/AcOH 5. Zn(OAc)_2_ (cat.), 170 °C, 12 h/quinoline	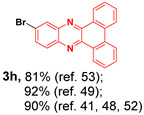	[41,48,49,52,53]
8		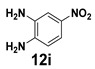	1. **Fe_3_O_4_@APTES@isatin-SO_3_H**, MNR catalyst, rt, 20 min/EtOH 2. ionic liquid **[BBSA-DBN][HSO_4_]** (3 mol%), 80 °C, 30 min/EtOH 3. SiO_2_/**[SEP]Cl**, 65 °C, 2 min/EtOH 4. HOOCCH_2_C(=CH_2_)COOH, rt, 1 h/H_2_O 5. **[DABCO](SO_3_H)_2_CoCl_4_** (20 mg), reflux, 30 min/H_2_O	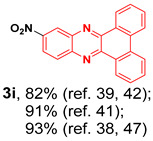	[38,39,41,42,47]
9		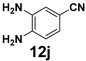	HOOCCH_2_C(=CH_2_)COOH, rt, 1 h/H_2_O	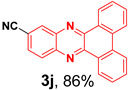	[42]
10		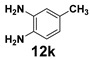	1. **Fe_3_O_4_@APTES@isatin-SO_3_H**, MNR catalyst, rt, 17 min/EtOH 2. ionic liquid **[BBSA-DBN][HSO_4_]** (3 mol%), 80 °C, 35 min/EtOH 3. HOOCCH_2_C(=CH_2_)COOH, rt, 1 h/H_2_O 4. mesoporous **TG-PSSA-co-ACA**, rt, 5 min/H_2_O 5. **[DABCO](SO_3_H)_2_CoCl_4_** (20 mg), reflux, 30 min/H_2_O	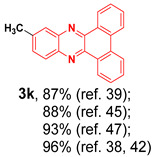	[38,39,41,42,47]
11	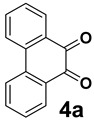	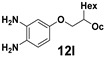 **Oc** = C_8_H_17_	75 °C, 16 h/EtOH	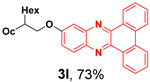	[54]
12		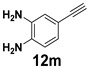	75 °C, 18 h/EtOH–CH_2_Cl_2_ (5:1)	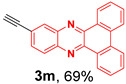	[51]
13		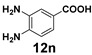	MW, 230 °C, 10 min/5% AcOH	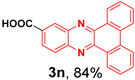	[55]
14		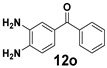	1. SiO_2_/**[SEP]Cl**, 65 °C, 15 min/EtOH 2. mesoporous **TG-PSSA-co-ACA**, rt, 5 min/H_2_O 3. 120 °C, 8 h/AcOH	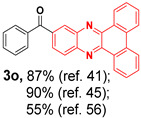	[41,45,56]
15		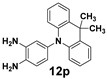	reflux, 12 h/*n*-BuOH	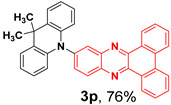	[57]
16		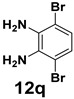	reflux, 12 h/AcOH	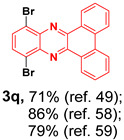	[49,58,59]
17		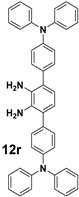	120 °C, 8 h/AcOH	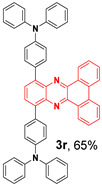	[60]
18		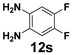	reflux, 12 h/*n*-BuOH	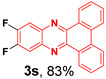	[61]
19		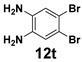	1. 80 °C, 4 h/AcOH–EtOH 2. reflux, 24 h/AcOH	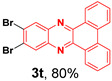	[62,63]
20		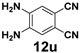	reflux, 8 h/AcOH	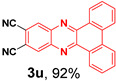	[64]
21		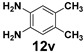	AcOH (cat.), reflux, 48 h/EtOH	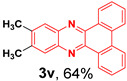	[65]
22	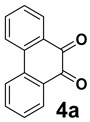	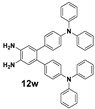	125 °C, 12 h/AcOH	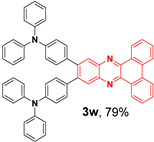	[66]
23		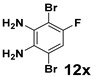	reflux, 12 h/AcOH	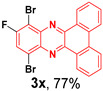	[59]
24		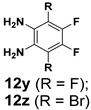	1. **R** = **F**: 125 °C, 12 h/AcOH 2. **R** = **Br**: reflux, 12 h/AcOH	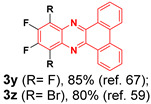	[59,67]
25		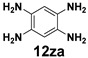	NEt_3_, reflux, 6 h/AcOH–EtOH (3:1)	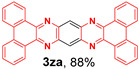	[46]
26		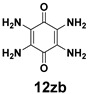	reflux, 6 h/AcOH–EtOH (3:1)	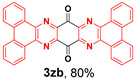	[68,69]
27		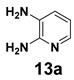	1. amorphous Fe nanoparticles, rt, 7 min/H_2_O 2. **CSA** (20 mol%), rt, 1 h 30 min/EtOH–H_2_O (1:1) 3. *nano*-Ca(IO)_3_, rt, 9 min/AcOH 4. reflux, 24 h/*i*-PrOH	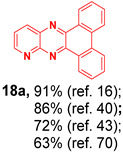	[16,40,43,70]
28		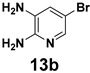	1. amorphous Fe nanoparticles, rt, 10 min/H_2_O 2. reflux, 8 h/*n*-BuOH	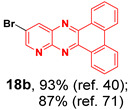	[40,71]
29		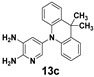	reflux, 12 h/*n*-BuOH	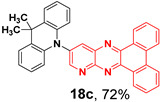	[57]
30		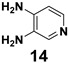	SiO_2_/**[SEP]Cl**, 65 °C, 15 min/EtOH	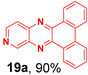	[41]
31		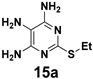	110 °C, 5 h/DMF	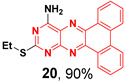	[72]
32		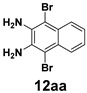	reflux, 6 h/AcOH–EtOH (1:2)	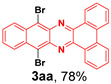	[73]
33	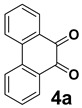	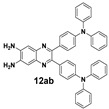	120 °C, 8 h/AcOH	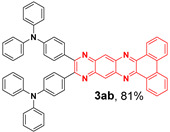	[74]
34		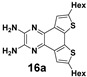	reflux, 12 h/AcOH–EtOH (1:1)	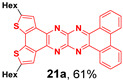	[75]
35		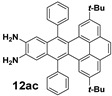	80 °C, 24 h/AcOH–CHCl_3_ (1:1)	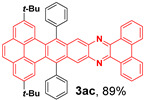	[76]
36		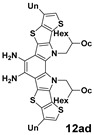 **Oc** = C_8_H_17_ **Un** = C_11_H_23_	100 °C, 12 h/AcOH–EtOH (1:1)	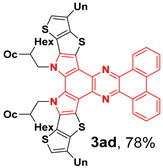	[77]
37	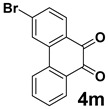	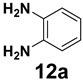	reflux, 2 h/AcOH	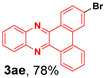	[78]
38		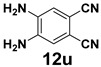	reflux, 12 h/AcOH	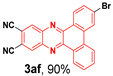	[79,80]
39	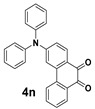	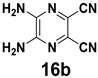	reflux, 24 h/AcOH	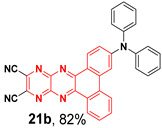	[81]
40	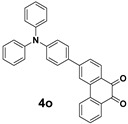	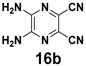	reflux, 24 h/AcOH	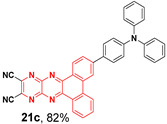	[81]
41	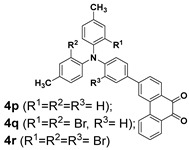	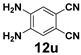	1. **R^1^** = **R^2^** = **R^3^** = H, 36% HCl, 40 °C, 48 h/EtOH–THF (1:1) 2. **R^1^** = **R^2^** = **Br**, **R^3^** = **H**, HCl (cat.), rt, 12 h/EtOH–THF (1:1) 3. **R^1^** = **R^2^** = **R^3^** = **Br**, HCl (cat.), rt, 12 h/EtOH–THF (1:1)	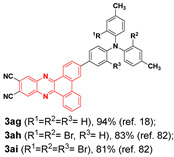	[18]
42	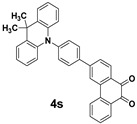	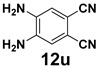	36% HCl, 40 °C, 48 h/EtOH–THF (1:1)	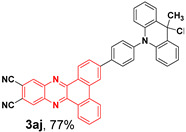	[18]
43	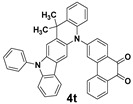	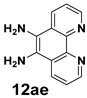	reflux, 12 h/*n*-BuOH	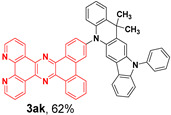	[82]
44	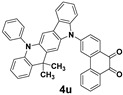	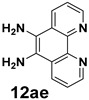	reflux, 12 h/*n*-BuOH	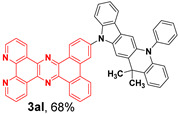	[82]
45	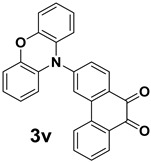	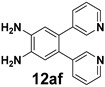	reflux, 12 h/*n*-BuOH	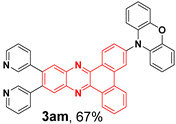	[83]
46		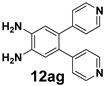	reflux, 8 h/AcOH	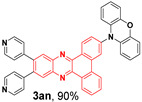	[84]
47		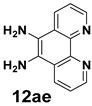	reflux, 12 h/*n*-BuOH	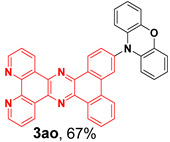	[83]
48	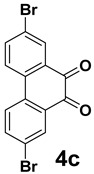	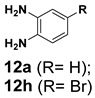	1. **R** = **H**, TsOH (cat.), reflux, 5 h/EtOH 2. **R** = **H**, 130 °C, 4 h/AcOH 3. **R** = **Br**, 130 °C, 4 h/AcOH	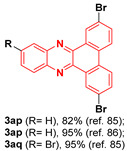	[85,86]
49		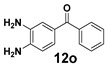	reflux, 6 h/AcOH–EtOH	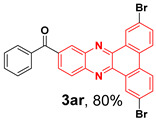	[87]
50		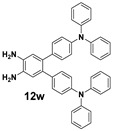	125 °C, 12 h/AcOH	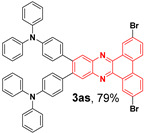	[66]
51	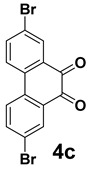	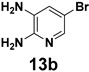	AcOH (cat.), reflux, 1.5 h/EtOH	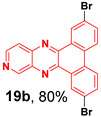	[88]
52		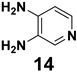	AcOH (cat.), reflux, 5 h/EtOH	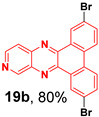	[89]
53		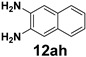	reflux, 6 h/AcOH–EtOH (1:2)	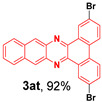	[73]
54		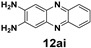	reflux, 24 h/AcOH	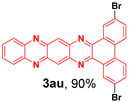	[90]
55	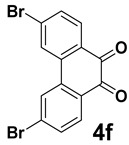 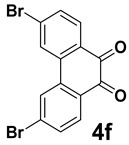	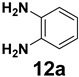	1. reflux, 2 h/AcOH 2. 80 °C, 4 h/AcOH–EtOH 3. reflux, 8 h/AcOH 4. TsOH (cat.), reflux, 2 h/AcOH	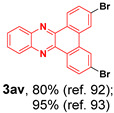	[62,78,91,92]
56		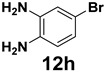	1. reflux, 3 h/AcOH 2. 110 °C, 6 h/AcOH	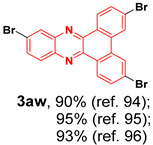	[93,94,95]
57		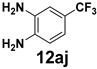	110 °C, 24 h/AcOH	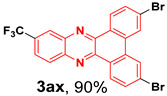	[96]
58		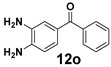	1. reflux, 8 h/AcOH 2. reflux, 3 h/AcOH	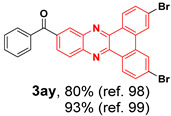	[97,98]
59		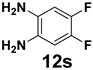	1. 80 °C, 2 h/EtOH 2. reflux, 12 h/AcOH	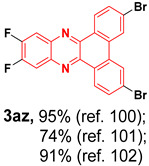	[99,100,101]
60	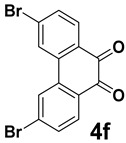 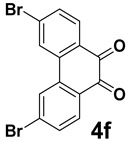	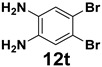	1. reflux, 3 h/AcOH 2. reflux, 6 h/AcOH 3. reflux, 8 h/AcOH 4. reflux, 10 h/EtOH	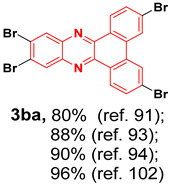	[91,93,94,102]
61		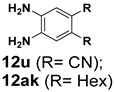	reflux, 8 h/AcOH	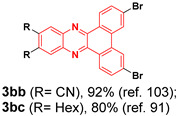	[91,103]
62		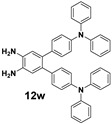	125 °C, 24 h/AcOH	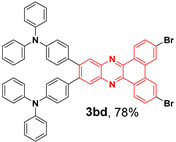	[104]
63		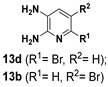	1. **R^1^** = **Br**, **R^2^** = **H**: reflux, 6 h/EtOH 2. **R^1^** = **H**, **R^2^** = **Br**: AcOH (cat.), reflux, 1 h 30 min/EtOH 3. **R^1^** = **H**, **R^2^** = **Br**: reflux, 6 h/EtOH	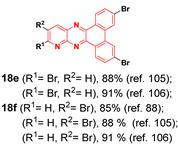	[88,105,106]
64		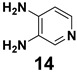	AcOH (cat.), reflux, 5 h/EtOH	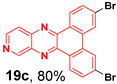	[89]
65		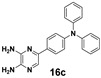	reflux, 24 h/AcOH	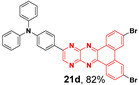	[81]
66		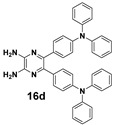	reflux, 24 h/AcOH	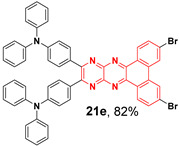	[81]
67		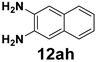	reflux, 6 h/AcOH–EtOH (1:2)	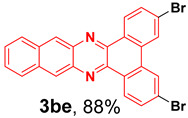	[73]
68	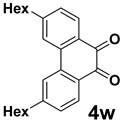	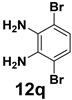	reflux, 12 h/AcOH	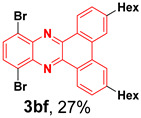	[58]
69	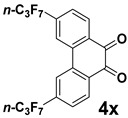	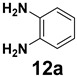	35 °C, 10 h/AcOH–EtOH (1:3)	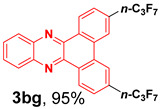	[107]
70	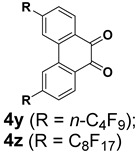	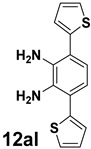	90 °C, 24 h/AcOH–EtOH (1:3)	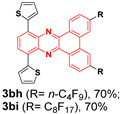	[108]
71	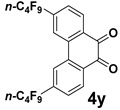	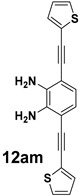	90 °C, 24 h/AcOH–EtOH (1:3)	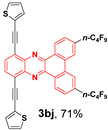	[108]
72	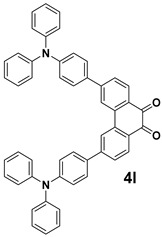	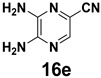	reflux, 24 h/AcOH	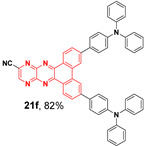	[81]
73		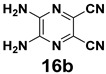	reflux, 24 h/AcOH	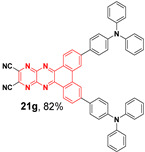	[81]
74	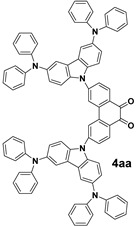	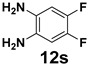	reflux, 14 h/*n*-BuOH	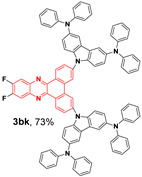	[61]
75	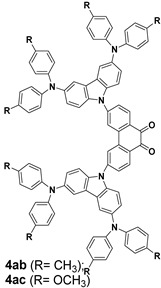	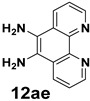	140 °C, 14 h/*n*-BuOH	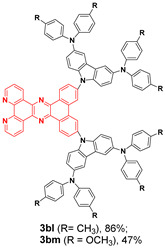	[109]
76	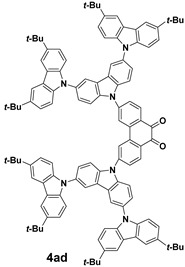	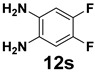	reflux, 14 h/*n*-BuOH	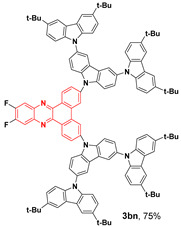	[61]
77	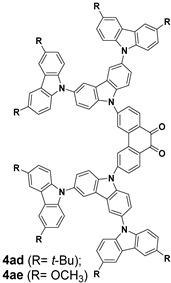	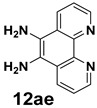	140 °C, 24 h/*n*-BuOH	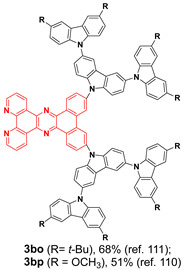	[109,110]
78	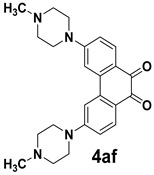	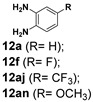	AcOH (cat.), reflux, 24 h/EtOH	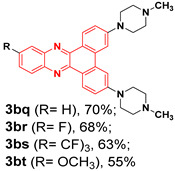	[111]
79		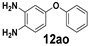	AcOH (cat.), reflux, 24 h/EtOH	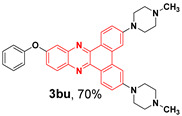	[111]
80	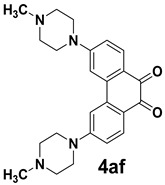	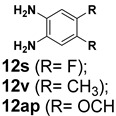	AcOH (cat.), reflux, 24 h/EtOH	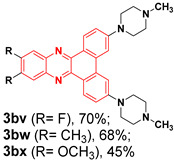	[111]
81		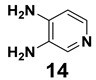	AcOH (cat.), reflux, 24 h/EtOH	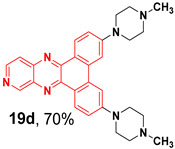	[111]
82		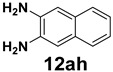	AcOH (cat.), reflux, 24 h/EtOH	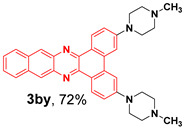	[111]
83	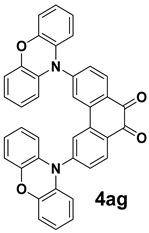	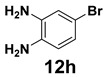	100 °C, 8 h/AcOH	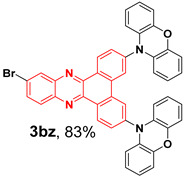	[112]
84		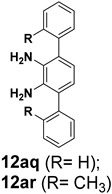	reflux, 12 h/*n*-BuOH	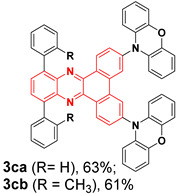	[113]
85		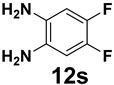	100 °C, 8 h/AcOH	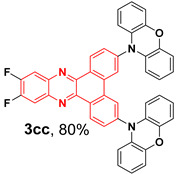	[112]
86		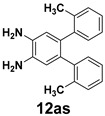	reflux, 12 h/*n*-BuOH	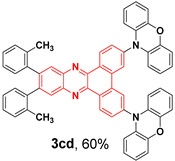	[113]
87	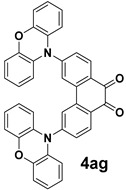	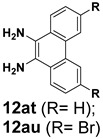	105 °C, 8 h/*t*-BuOH	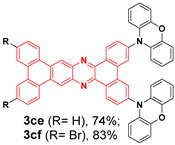	[114]
88	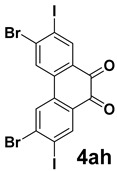	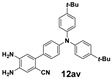	130 °C, 4 h/AcOH	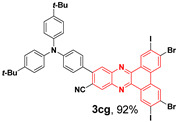	[115]
89	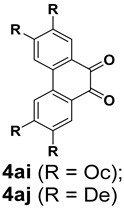 **Oc** = **C_8_H_17_** **De** = **C_10_H_21_**	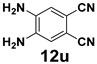	reflux, 3 h/AcOH–toluene	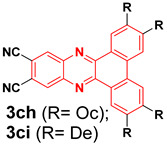	[116]
90	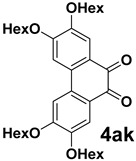	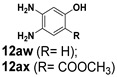	AcOH (cat.), reflux, 16 h/EtOH	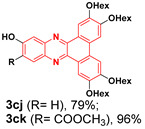	[117]
91	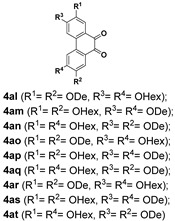	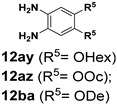	NaOAc, reflux, 12 h/EtOH	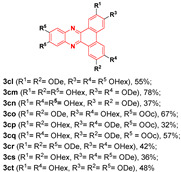	[118]
92	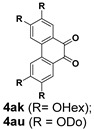	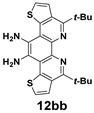	reflux, 72 h/AcOH–EtOH (1:5)	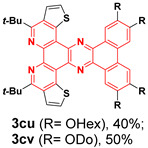	[119]
93	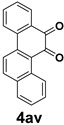	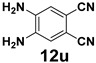	reflux, 63 h/EtOH	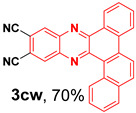	[120]
94	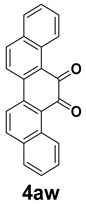	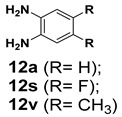	reflux, 24 h/AcOH	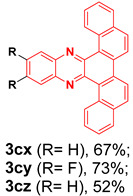	[121]
95		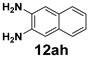	reflux, 24 h/AcOH	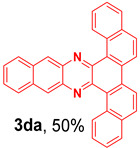	[121]
96	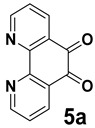 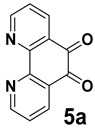 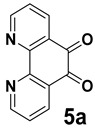	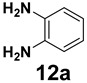	1. reflux, 3 h/EtOH 2. rt, 3 h/MeOH 3. 50 °C, 5 h/EtOH 4. reflux, 4 h/DMF 5. reflux, 10 h/MeOH 6. reflux, 8 h/EtOH	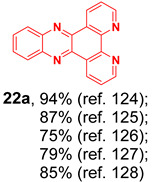	[122,123,124,125,126,127,128]
97		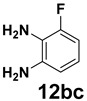	reflux, 4 h/EtOH	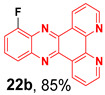	[129,130]
98		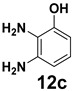	1. reflux, 3 h/EtOH 2. reflux, 4 h/EtOH	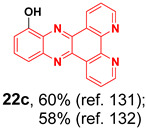	[130,131]
99		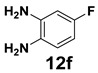	1. reflux, 4 h/EtOH 2. rt, 3 h/MeOH 3. reflux, 4 h/AcOH	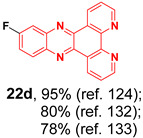	[122,123,124,132,133]
100		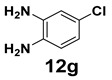	1. rt, 3 h/MeOH 2. reflux, 6 h/EtOH	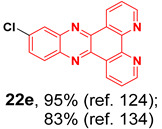	[124,134]
101		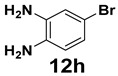	1. rt, 2 h/CH_2_Cl_2_ 2. reflux, 4 h/EtOH 3. reflux, 8 h/EtOH	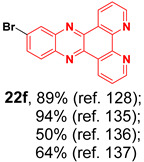	[128,135,136,137]
102		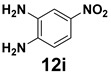	1. reflux, 4 h/EtOAc–MeOH (7:1) 2. reflux, 4 h/EtOH 3. reflux, 8 h/EtOH	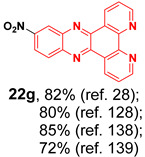	[28,128,138,139]
103		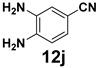	1. reflux, 4 h/AcOH 2. reflux, 6 h/MeOH 3. reflux, 10 h/MeOH 4. reflux, 24 h/AcOH–EtOH (1:1)	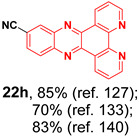	[127,133,140,141]
104		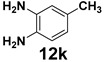	reflux, 4 h/AcOH	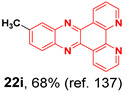	[137,142]
105		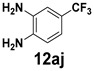	1. reflux, 4 h/EtOH 2. rt, 3 h/MeOH	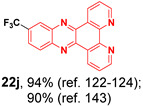	[122,123,124,143]
106		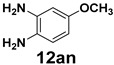	1. reflux, 4 h/EtOH 2. reflux, 10 h/MeOH 3. reflux, 8 h/EtOH	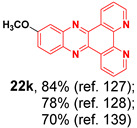	[127,128,139]
107		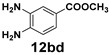	1. reflux, 4 h/AcOH 2. reflux, 24 h/EtOH	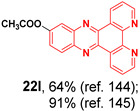	[144,145]
108		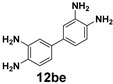	reflux, 2 h/AcOH	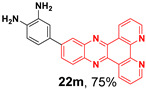	[146]
109		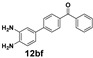	AcOH (cat.), 100 °C, 12 h/EtOH	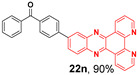	[147]
110		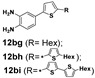	reflux, 15 h/EtOH	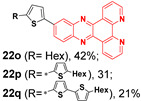	[148]
111		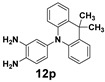	reflux, 12 h/*n*-BuOH	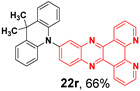	[57]
112		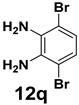	Et_3_N (cat.), reflux, 3 h/EtOH	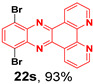	[149]
113		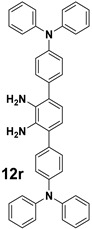	100 °C, 12 h/AcOH	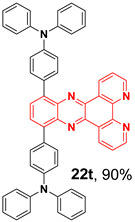	[150]
114		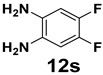	1. reflux, 3 h/EtOH	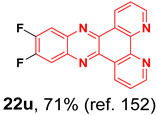	[151]
115		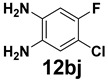	rt, 3 h/MeOH	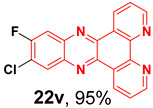	[124]
116		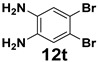	reflux/EtOH	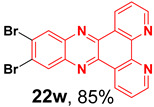	[152]
117		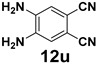	1. AcOH (cat.), reflux, 2 h/EtOH 2. reflux, 8 h/AcOH	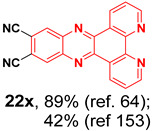	[64,153]
118		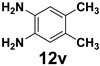	1. rt, 3 h/MeOH 2. reflux, 4 h/EtOH 3. reflux, 2 h/EtOH	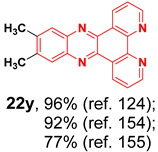	[124,154,155]
119		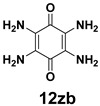	reflux, 6 h/AcOH–EtOH (3:1)	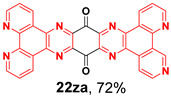	[68,69]
120	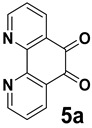	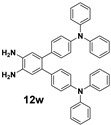	60 °C, 8 h/CH_2_Cl_2_	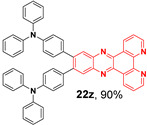	[156]
121		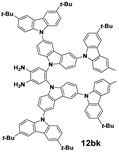	reflux, 12 h/*n*-BuOH	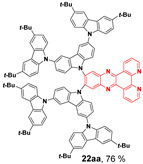	[110]
122		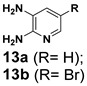	**R** = **H**: 1. reflux, 1 h 30 min/MeOH 2. AcNH_4_ (cat.), reflux, 4 h/AcOH **R** = **Br**: AcNH_4_ (cat.), reflux, 4 h/AcOH	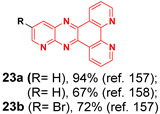	[157,158]
123		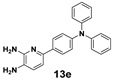	reflux, 12 h/AcOH	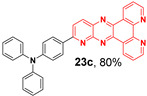	[159]
124		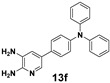	reflux, 12 h/AcOH	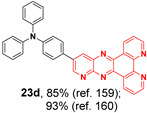	[159,160]
125		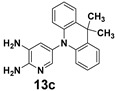	reflux, 12 h/*n*-BuOH	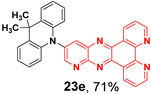	[57]
126		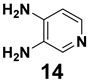	AcNH_4_ (cat.), reflux, 4 h/AcOH	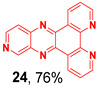	[158]
127		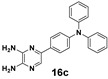	120 °C, 12 h/AcOH	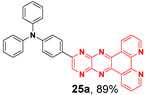	[160]
128		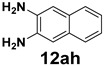	rt, 3 h/MeOH	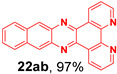	[124]
129		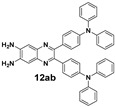	120 °C, 8 h/AcOH	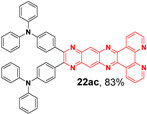	[74]
130	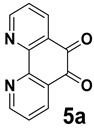	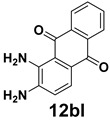	reflux, 9 h/EtOH	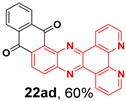	[161]
131		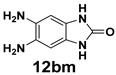	1. reflux, 15 min/EtOH 2. reflux, 1 h/EtOH	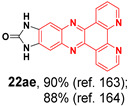	[162,163]
132		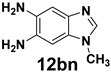	reflux, 2 h/EtOH	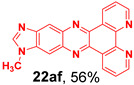	[164]
133		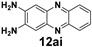	CF_3_COOH, reflux, 14 h/EtOH	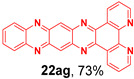	[165]
134		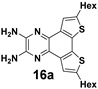	reflux, 12 h/AcOH–EtOH (1:1)	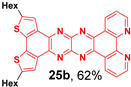	[75]
135		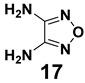	1. reflux, 5 h/AcOH 2. reflux, 4 h/EtOH	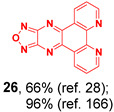	[28,166]
136	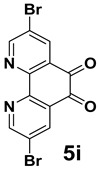	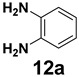	reflux, 12 h/EtOH	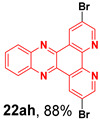	[167]
137		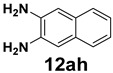	reflux, 16 h/EtOH	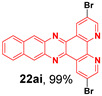	[167]
138	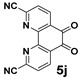	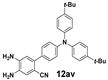	125 °C, 12 h/AcOH	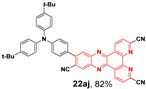	[168]
139	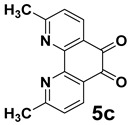	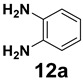	TsOH (cat.), reflux, 16 h/EtOH	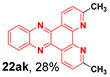	[27]
140		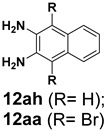	**R** = **H**: TsOH (cat.), reflux, 16 h/EtOH **R** = **Br**: 80 °C, 16 h/AcOH	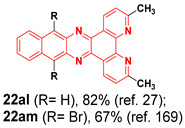	[27,169]
141	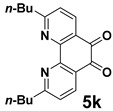	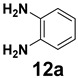	reflux, 6 h/EtOH	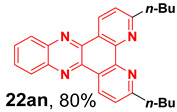	[170]
142	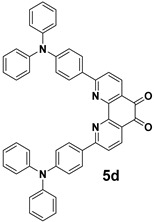	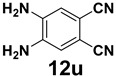	120 °C, 12 h/AcOH	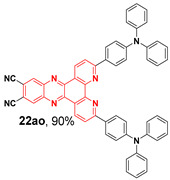	[171]
143	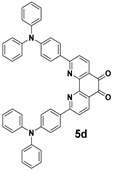	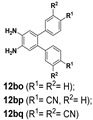	120 °C, 12 h/AcOH	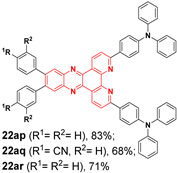	[172]
144	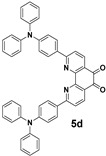	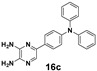	120 °C, 10 h/AcOH	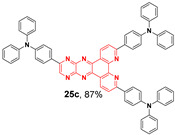	[160]
145	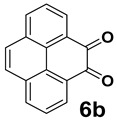	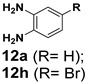	**R** = **H**: reflux, 2 h/AcOH **R** = **Br**: Zn(OAc)_2_ (cat.), 170 °C, 12 h/quinoline	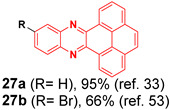	[33,53]
146		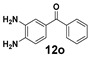	reflux, 12 h/AcOH	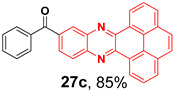	[173]
147		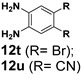	**R** = **Br**: 120 °C, 24 h/AcOH **R** = **CN**: 80 °C, 19 h/AcOH–EtOH (1:1)	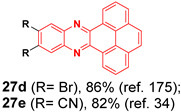	[34,174]
148		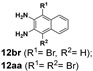	80 °C, 15 h/AcOH–EtOH (1:1)	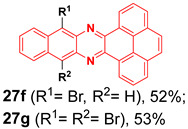	[175]
149	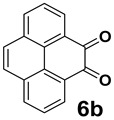	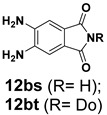 **Do** = C_12_H_25_	AcOH (cat.), reflux, 24 h/EtOH	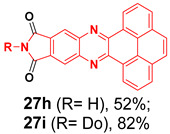	[176]
150		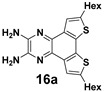	reflux, 12 h/AcOH–EtOH (1:1)	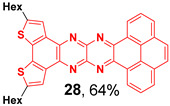	[75]
151		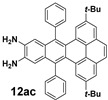	80 °C, 24 h/AcOH–CHCl_3_ (1:1)	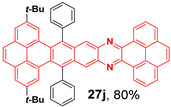	[76]
152	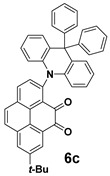	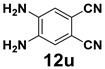	reflux, 8 h/AcOH	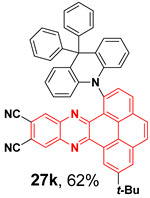	[35]
153	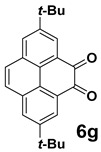	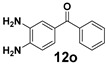	reflux, 12 h/AcOH	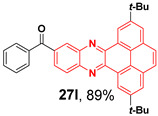	[173]
154		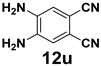	reflux, 24 h/AcOH	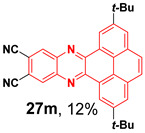	[177]
155	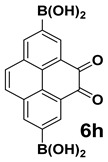	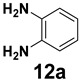	100 °C, 192 h/DMSO	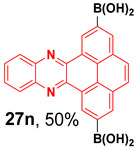	[178]
156		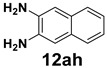	100 °C, 192 h/DMSO	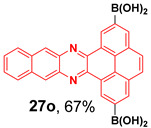	[178]
157	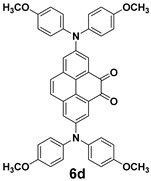	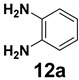	80 °C, 15 h/AcOH–EtOH (1:1)	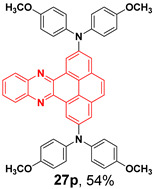	[36]
158	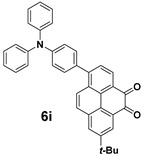	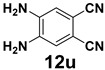	reflux, 12 h/AcOH	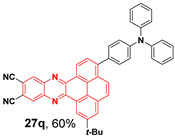	[179]
159	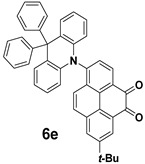	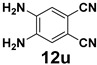	reflux, 8 h/AcOH	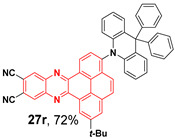	[35]
160	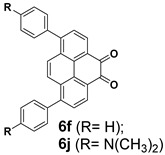	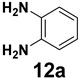	reflux, 2 h/AcOH	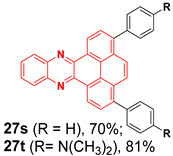	[33]
161	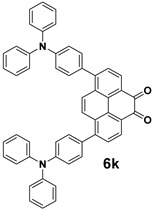	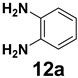	reflux, 12 h/AcOH–EtOH (1:1)	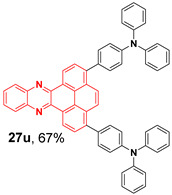	[32]
162	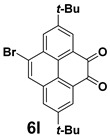	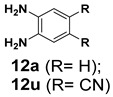	reflux, 3 h/AcOH	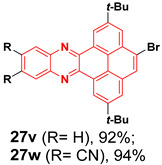	[180]
163	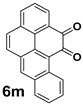	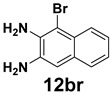	80 °C, 15 h/AcOH–EtOH (1:1)	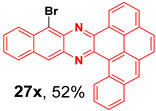	[175]
164	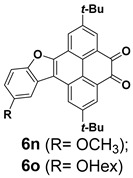	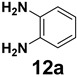	75 °C, 24 h/AcOH–CHCl_3_ (1:1)	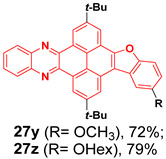	[181]

**Table 3:** Structures of some polycycles and photovoltaic parameters of the solar cells based on them.

Structure	*V*_oc_, V	*J*_sc_, mA·cm^−1^	*FF*,%	*η*, %	Ref.
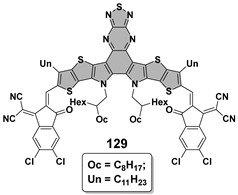	0.859	27.72	80.12	19.09	[202]
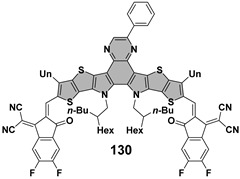	0.889	25.80	79.06	18.14	[206] [206]
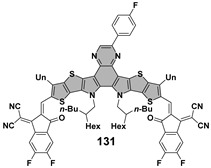	0.872	26.40	79.17	18.21	
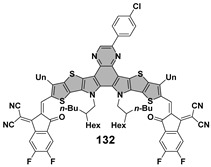	0.880	26.36	78.24	18.19	
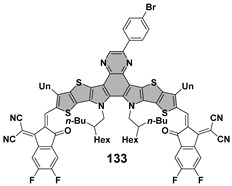	0.886	25.80	81.45	18.62	
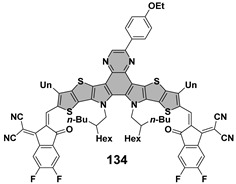	0.904	24.48	75.84	16.79	

## Data Availability

The original research data is available upon request.
